# Oligonucleotide correction of an intronic *TIMMDC1* variant in cells of patients with severe neurodegenerative disorder

**DOI:** 10.1038/s41525-021-00277-7

**Published:** 2022-01-28

**Authors:** Raman Kumar, Mark A. Corbett, Nicholas J. C. Smith, Daniella H. Hock, Zoya Kikhtyak, Liana N. Semcesen, Atsushi Morimoto, Sangmoon Lee, David A. Stroud, Joseph G. Gleeson, Eric A. Haan, Jozef Gecz

**Affiliations:** 1 Adelaide Medical School and the Robinson Research Institute, University of Adelaide, Adelaide, 5000 SA Australia; 2 Department of Neurology, Women’s and Children’s Health Network, Adelaide, 5006 SA Australia; 3 Department of Biochemistry and Pharmacology, Bio21 Molecular Science and Biotechnology Institute, University of Melbourne, Parkville, 3052 VIC Australia; 4 Dame Roma Mitchell Cancer Research Laboratories, Adelaide Medical School, University of Adelaide, Adelaide, 5000 SA Australia; 5 Neurogenetics Laboratory, Institute for Genomic Medicine and Departments of Neurosciences and Paediatrics, University of California, San Diego, 92093 CA USA; 6 Murdoch Children’s Research Institute, The Royal Children’s Hospital, Parkville, 3052 VIC Australia; 7 Adult Genetics Unit, Royal Adelaide Hospital and Faculty of Health and Medical Sciences, University of Adelaide, Adelaide, 5000 SA Australia

**Keywords:** Molecular medicine, Clinical genetics

## Abstract

*TIMMDC1* encodes the Translocase of Inner Mitochondrial Membrane Domain-Containing protein 1 (TIMMDC1) subunit of complex I of the electron transport chain responsible for ATP production. We studied a consanguineous family with two affected children, now deceased, who presented with failure to thrive in the early postnatal period, poor feeding, hypotonia, peripheral neuropathy and drug-resistant epilepsy. Genome sequencing data revealed a known, deep intronic pathogenic variant *TIMMDC1* c.597-1340A>G, also present in gnomAD (~1/5000 frequency), that enhances aberrant splicing. Using RNA and protein analysis we show almost complete loss of TIMMDC1 protein and compromised mitochondrial complex I function. We have designed and applied two different splice-switching antisense oligonucleotides (SSO) to restore normal *TIMMDC1* mRNA processing and protein levels in patients’ cells. Quantitative proteomics and real-time metabolic analysis of mitochondrial function on patient fibroblasts treated with SSOs showed restoration of complex I subunit abundance and function. SSO-mediated therapy of this inevitably fatal TIMMDC1 neurologic disorder is an attractive possibility.

## Introduction

The pace, sensitivity, and accuracy of identifying variants, both in known and new disease genes, has improved dramatically during the last few years^1^. Further, rapid improvements in tools for combined analysis of genome sequencing and RNAseq data from patient-derived cells or disease-relevant tissues have been particularly effective in identifying disease variants with splicing or gene-regulatory effects^2–4^. However, clarifying the functional and clinical significance of the rapidly increasing number of detected gene variants remains a huge challenge that trails advances in variant identification.

In 2015 we reported a consanguineous family with two siblings with an infantile-onset neurodegenerative disorder manifesting a predominant sensorimotor axonal neuropathy, optic atrophy, and cognitive deficit. Using identity by descent mapping to 3q13.13-21.1 and family-based whole-genome sequencing, we identified at that time a unique homozygous syntaxin-binding protein 5-like missense variant (NM_001308330.2 (*STXBP5L*):c.3055G>A (p.Val1019Ile), ClinVar: VCV000266019.1)^5^. Combined genetic and molecular functional evidence was consistent with the STXBP5L p.Val1019Ile variant being the likely cause of the disorder^5^. However, the variant has uncertain clinical significance by current American College of Medical Genetics and Genomics and the Association for Molecular Pathology guidelines^6^.

*TIMMDC1*, located at chromosome 3q13.33 1.4 Mb centromeric to *STXBP5L*, encodes the Translocase of Inner Mitochondrial Membrane Domain-Containing protein 1 (TIMMDC1) assembly factor of complex I, the largest complex of the electron transport chain (ETC). TIMMDC1 is a multi-pass membrane protein found within the inner mitochondrial membrane and implicated in the biogenesis of core complex I subunit ND1, one of the 13 proteins encoded by mitochondrial DNA^7–9^. Three unrelated children with clinical features similar to those of our family were reported by Kremer et al. to have a homozygous c.596+2146A>G *TIMMDC1* variant, currently referred to as c.597-1340A>G^4^. This variant results in aberrant splicing of the *TIMMDC1* transcript, with insertion of a poison exon that introduces a frameshift leading to a premature stop codon p.Gly199_Thr200ins5* and thus nonsense-mediated decay (NMD) of the aberrant transcript in patient cells. Consequently, the TIMMDC1 protein is undetectable in the patient cells^4^, resulting in compromised complex I function. More recently, two affected children with compound heterozygous (c.385C>T, p.Arg129*; 596+2146A>G, p.Gly199_Thr200ins5*) *TIMMDC1* variants were published^10^. The phenotypic similarity between these reported individuals and the siblings we described in 2015^5^, together with the fact that *TIMMDC1* maps within the identity by descent region, prompted us to re-examine the family. Combining RNAseq data from patients’ and parents’ fibroblasts with analysis by RNA splicing prediction tools of variants called from patient genome sequencing, we identified the same deep intronic *TIMMDC1* c.597-1340A>G variant as Kremer and colleagues^4^. Here, we report the genetic, expression and functional data showing that the *TIMMDC1* c.597-1340A>G variant is the primary cause of the disorder in this family. We also show restoration of *TIMMDC1* mRNA, protein, an abundance of complex I subunits and mitochondrial function using splice-switching antisense oligonucleotides (SSO), suggesting potential for future therapeutic application of this approach for this severe, progressive neurological disorder.

## Results

### Clinical phenotype of the affected siblings is consistent with *TIMMDC1*-related disease and the wider spectrum of mitochondrial complex I dysfunction

The affected siblings, a brother and sister, children of first-cousin parents, had a mixed central and peripheral neurodegenerative disorder^5^. Their clinical features and those of other reported patients^4,10^ can be found in Supplementary Table 1.

The features observed in the siblings we described^5^ demonstrated considerable overlap with those of other children manifesting *TIMMDC1*-related disease^4,10^ and wider congruence with observed neurological and extra-neurological features of mitochondrial complex I deficiency^11–13^. Classic biochemical markers of perturbed mitochondrial oxidative phosphorylation have proved inconspicuous in all cases reported^4,5,10^; indeed, our sibship demonstrated normal venous and CSF lactate levels (III-2), with only occasional borderline elevations in blood lactate (in fewer than 15% of samples assayed), and a mild non-specific elevation of ketones and Krebs cycle metabolites on a single urinary organic acid profile (III-6), despite multiple blood and urine sampling points in each case. It is notable that, where documented, skeletal muscle respiratory chain enzymology confirmed isolated complex I deficiency; in our case, the muscle biopsy demonstrated significant adipose tissue deposition and the biochemical analyses proved inconclusive. Histopathological and ultrastructural features were similarly non-specific, reflecting features of muscle denervation only^4,5,10^ (Supplementary Table 1). Additionally, while a Leigh syndrome presentation has been described in one patient with *TIMMDC1* variant and characteristic MR features of bilateral T2 hyperintensities in the basal ganglia and/or brainstem are noted in two cases in T2-weighted sequences, such findings were not evident in the cases presented here, nor was elevated lactate present upon single-voxel proton spectroscopy (TE 31 and 144 ms) of the striatal grey matter (Supplementary Table 1).

### Identification of a pathogenic deep intronic *TIMMDC1* variant

We made a targeted review of all coding and non-coding variants within *TIMMDC1* according to our phenotype-driven hypothesis. The results concurred with our previous observations that there were no rare pathogenic variants within the coding or consensus splice sites of *TIMMDC1*^5^. Annotation of the variants against the ClinVar database detected a previously recorded pathogenic variant NM_016589.4(*TIMMDC1*):c.597-1340A>G VCV000429020.2; identical to the previously published NM_016589.4(*TIMMDC1*):c.596+2146A>G variant (now annotated as c.597-1340A>G)^4^ with genomic annotation NC_000003.11:g.119234712A>G. Splice prediction algorithms predicted only modest effects from this variant (Supplementary Table 2). Analysis of the reference sequence with Exonic Splicing Enhancer (ESE) finder^14^ located an ESE at +12 bp from the cryptic splice acceptor site of the 80 bp poison exon (Supplementary Fig. 1). This prediction remained unchanged by the c.597-1340A>G variant. Similarly, using the 80 bp reference and variant poison exon sequences as input for the EX-SKIP algorithm^15^ indicated that no novel ESE is created by the variant sequence.

### Segregation of the *TIMMDC1* c.597-1340A>G variant within the affected family

**Fig. 1:**
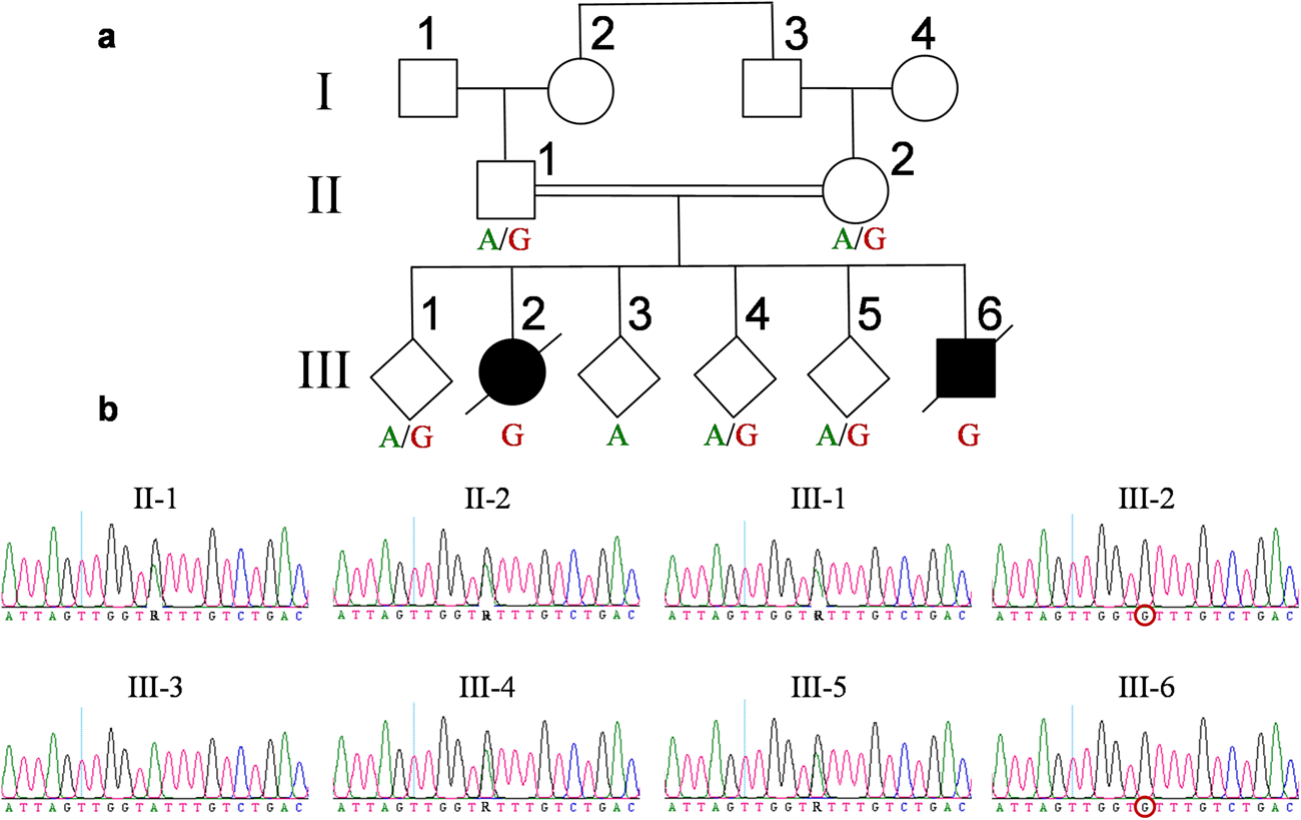
*TIMMDC1* rare intronic variant c.597-1340A>G segregates in the family as a recessive allele. **a** Family pedigree showing the consanguineous heterozygous (A/G) parents (II-1 and II-2), two affected homozygous (G) individuals (III-2 and III-6) and other unaffected heterozygous (A/G) or homozygous wild type (A) sibs. A slash symbol represents deceased individuals. **b** Segregation of *TIMMDC1* c.597-1340A>G variant. Sanger sequencing chromatograms show the presence or absence of the *TIMMDC1* variant in the family members. The *TIMMDC1* c.597-1340A>G homozygous variant in affected individuals III-2 and III-6 is marked with a red circle.

**Fig. 2:**
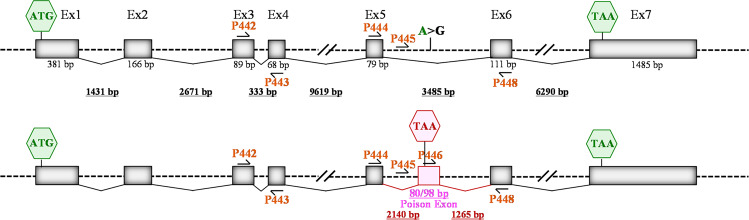
*TIMMDC1* intronic variant c.597-1340A>G inserts a poison exon between exons 5 and 6. Schematic structure of the *TIMMDC1* gene. Not to scale. Locations of the primers for generating data are presented in Figs. 6, 7 and poison exon between Exons 5 and 6 are shown.

We PCR amplified (using primers P445/P448; Supplementary Table 1; Figs. 1, 2) the genomic region flanking the *TIMMDC1* c.597-1340A>G variant using blood DNA from all family members (Fig. 1) and performed Sanger sequencing. The consanguineous parents (II-1 and II-2) were heterozygous (A/G), the two affected individuals (III-2 and III-6) homozygous (G/G) and the unaffected siblings either heterozygous (A/G) (III-1, III-4, III-5) or homozygous (A/A) (III-3) for the *TIMMDC1* alleles (Fig. 1). These results were consistent with autosomal recessive inheritance.

### The *TIMMDC1* c.597-1340A>G variant acts as a splice enhancer

**Fig. 3:**
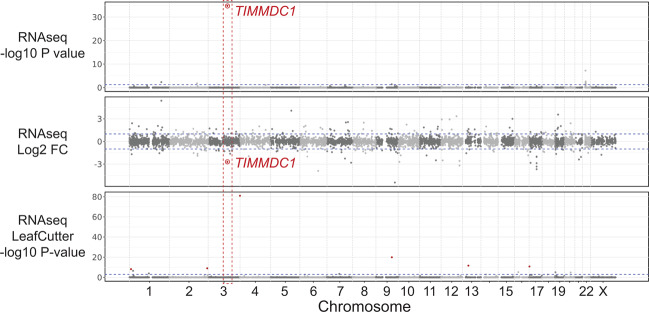
Only *TIMMDC1* gene is dysregulated in patient fibroblasts. Expression of only *TIMMDC1* gene that occurs within the previously mapped linkage interval is dysregulated (−2.7 Log_2_ fold) in patient fibroblasts compared with 107 control fibroblast cell lines analysed concurrently. Note that LeafCutter did not identify mis-splicing.

**Fig. 4:**
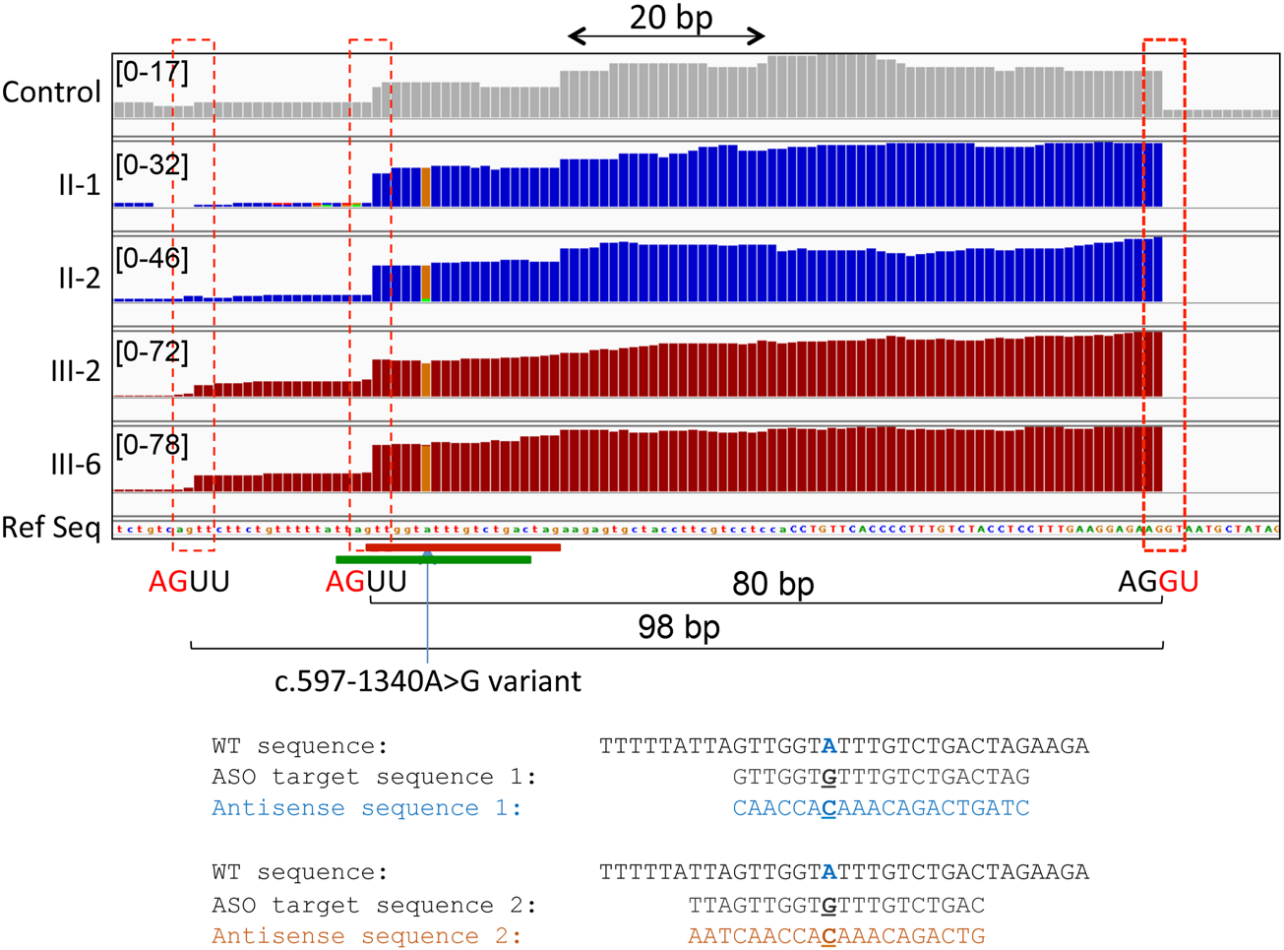
*TIMMDC1* c.597-1340A>G variant acts as a splice enhancer. Zoom in *TIMMDC1* exon showing two cryptic splice acceptor sites (AGUU) 5′ to the variant. Sequence reads from the parents (II-1 and II-2) and affected children (III-2 and III-6) contain this variant suggesting it drives mis-splicing. SSO1 and SSO2 antisense oligonucleotide target sequences are included and marked with red and green bars, respectively.

**Fig. 5:**
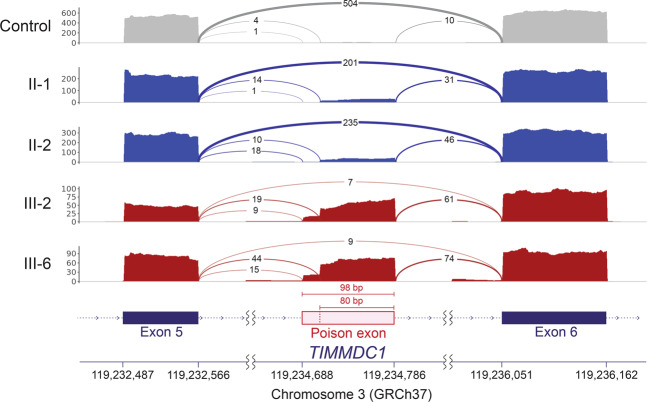
*TIMMDC1* c.597-1340A>G variant enhances alternative splicing across exons 5 and 6. *TIMMDC1* c.597-1340A>G variant-induced aberrant splicing across exons 5 and 6 inserts higher levels of a poison exon in the affected individuals (III-2 and III-6) than in both parents (II-1 and II-2) and that is almost absent in control mRNAs. Sashimi plots showing *TIMMDC1* mRNA read density maps for parents (blue), two affected children (red) and an unrelated healthy control (grey). The new poison exon is shown in pink. Note number of mRNA reads are reduced in the homozygous patients (III-2 and III-6) and heterozygous parents (II-1 and II-2) compared to the unrelated healthy control.

RNAseq data analysis identified *TIMMDC1* as an expression outlier in a cohort of 200 fibroblast samples with log_2_ fold change −2.71 and adjusted *p*-value 1.68 × 10^−35^; however, no outlier splicing events were detected by LeafCutter (Fig. 3). Manual inspection of the aligned RNAseq data of the parents, the two affected children and an unrelated individual showed two cryptic splice acceptor sites (AGUU) 5 bp (3:119,234,706) and 23 bp (3:119,234,688) 5′, and a cryptic splice donor site 74 bp (3:119,234,786) 3′ of the *TIMMDC1* c.597-1340A>G intronic variant. Comparison of the total number of sequence reads mapped to these junctions suggested that the *TIMMDC1* c.597-1340A>G variant acts as a splicing enhancer, causing aberrant splicing that leads to insertion of an 80 bp, or to a lesser extent, a 98 bp poison exon between Exon 5 and Exon 6 (Figs. 4 and 5). This is also clear from the Sashimi plots showing significantly higher *TIMMDC1* mRNA read density for the poison exon in the affected individuals homozygous for the intronic variant (average 89.4%: III-2 and III-6) than the parents heterozygous for the variant (average 14.9%; II-1 and II-2) and in the unrelated homozygous healthy control (1.9%) (Fig. 5).

### The *TIMMDC1* c.597-1340A>G variant inserts predominantly an 80 bp poison exonic sequence between Exon 5 and Exon 6 of *TIMMDC1* mRNAs of the affected individuals

**Fig. 6:**
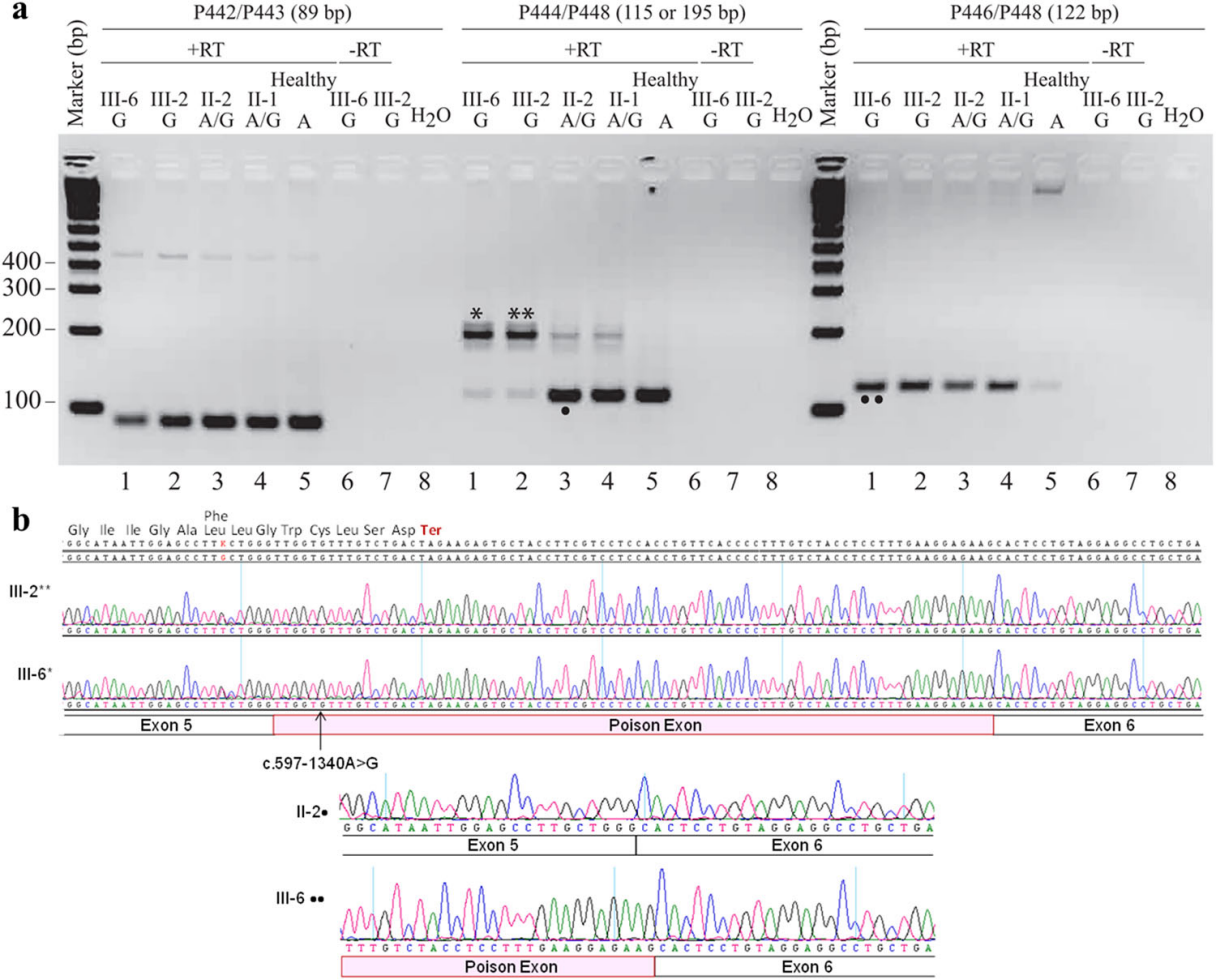
*TIMMDC1* c.597-1340A>G variant inserts an 80 bp poison exonic sequence between Exon 5 and 6 in *TIMMDC1* mRNAs of the affected individuals. **a** Agarose gel showing semi-quantitative RT-PCR amplicons from normally and alternatively spliced mRNAs from two affected (lanes 1 and 2), carrier parents (lanes 3 and 4) and an unrelated healthy control (lane 5) fibroblast RNAs. Minus RT reactions of the affected fibroblast RNAs (lanes 6 and 7) were also included. Left panel: Control PCR products from primers (P442/P443) located in Exon 3 and 4 showing amplification from the unaltered mRNA region, middle panel: PCR products from primers (P444/448) located in Exon 5 and 6 showing amplification of mRNAs with (lanes 1 and 2) and without (lanes 3–5) poison exon, and right panel: PCR products from primers (P446 located within poison exon and P448 within Exon 6) that specifically amplify mRNAs with poison exon sequence. Note that levels of mRNA with poison exon sequences in the affected individuals and parents (carrying the *TIMMDC1* variant; lanes 1–4) are higher than the unrelated control (lane 5) that has low level of aberrant splicing that generates the mRNAs with poison exon sequences. See Fig. 2 for primer location. **b** Chromatograms showing mRNA sequences with an 80 bp poison exon in fibroblasts of the two affected individuals (see sequence of III-6*, III-2** and III-6•• bands from the gel in a). Parent II-2• band sequence, as expected, showed the absence of poison exon.

We performed semi-quantitative RT-PCR analysis that allowed simultaneous assessment of relative levels of *TIMMDC1* transcripts with or without the poison exon (Fig. 6). When PCR was performed using primers located within Exon 5 and Exon 6 (P444/P448; see Fig. 2), the two affected individuals (III-2 and III-6) showed a predominant 195 bp amplification product representing the *TIMMDC1* transcript with the poison exon, and low levels of a 115 bp product from the normally spliced transcript (Fig. 6a; middle panel, lanes 1 and 2). The parents had both the 115 bp normally spliced and low levels (possibly due to PCR bias) of the 195 bp aberrantly spliced transcript (Fig. 6a; middle panel, lanes 3 and 4). The 195 bp band was undetectable in an unrelated healthy control individual who did not have the c.597-1340A>G allele (Fig. 6a; middle panel, lane 5). No amplification was observed from non-reverse transcribed RNA (Fig. 6a; middle panel, lanes 6 and 7). That mRNA 5′ to the aberrantly spliced sequence was unaltered by the intronic variant was checked by RT-PCR using primers (P442/P443) located within Exon 3 and Exon 4 (Fig. 6a; left panel). We also performed RT-PCR with a poison exon-specific primer (P446) to determine the levels of *TIMMDC1* transcript with the poison exon in patient, parent, and control fibroblasts. The data showed higher levels of poison transcript in patients than parents, and very low levels in the healthy control (Fig. 6a), suggesting the presence of a low level of aberrant splicing events from the cryptic donor site located at 3:119,234,786 even in the fibroblasts lacking the c.597-1340A>G allele. Sanger sequencing of the middle predominant PCR band (from III-6* and III-2**; Fig. 6a) confirmed the presence of an 80 bp poison exon sequence in the aberrantly spliced *TIMMDC1* transcript (Fig. 6b). This result is consistent with the RNAseq data presented in Fig. 4.

### *TIMMDC1* SSOs restore normal splicing in patient fibroblasts with the c.597-1340A>G variant

**Fig. 7:**
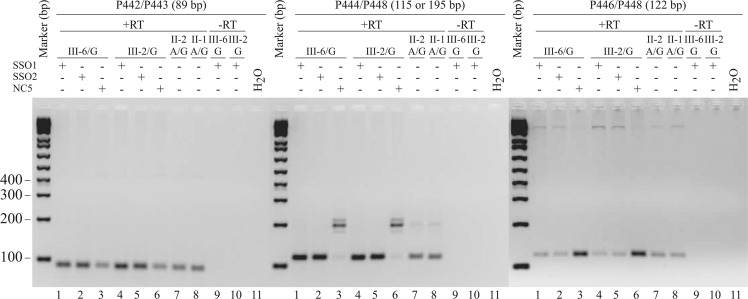
*TIMMDC1* SSO1 and SSO2 antisense oligonucleotides restore normal splicing in the c.597-1340A>G affected fibroblasts. Agarose gels showing semi-quantitative RT-PCR amplicons of normally and alternatively spliced mRNAs in the SSO1, SSO2 or non-specific control NC5 antisense oligonucleotides treated affected (III-6 and III-2; lanes 1–6) and untreated parent (II-1 and II-2) fibroblasts (lanes 7 and 8). Minus RT reactions of the affected fibroblast RNAs (lanes 9 and 10) are also shown. Left panel: Control PCR products from primers (P442/P443) located in Exon 3 and 4 showing amplification from the unaltered mRNA region. The gel showed increased levels of normal *TIMMDC1* mRNA in SSO1 and SSO2 (lanes 1, 2 and 4, 5) compared to NC5 (lanes 3, 6) treated affected or untreated (lanes 7, 8) parent fibroblasts. Middle panel: PCR products from primers (P444/448) located in Exon 5 and 6 showing increased levels of normally spliced *TIMMDC1* mRNA in SSO1 and SSO2 (lanes 1, 2 and 4, 5) compared to NC5 (lane 3, 6 with predominantly poison exon containing mRNA) treated affected or untreated (lanes 7 and 8 with predominantly normal mRNA) parent fibroblasts. Right panel: PCR products from primers (P446 located within poison exon and P448 within Exon 6) that specifically amplify mRNAs with poison exon sequence. Levels of poison exon containing mRNA is reduced in SSO1 and SSO2 treated (lanes 1, 2 and 4, 5) compared to NC5 (lanes 3 and 6) treated affected fibroblasts but comparable to untreated (lanes 7 and 8) parent fibroblasts.

We designed two overlapping *TIMMDC1* SSOs targeting the acceptor site of the major cryptic exon and the A>G variant sequence located close to this splice site to explore the possibility of inhibiting aberrant splicing caused by the deep intronic variant (see Fig. 4; SSO sequences in Table S1). We performed semi-quantitative RT-PCR analysis on fibroblasts transfected with two *TIMMDC1* SSOs targeting the variant sequence or non-targeting control NC5^16^ and determined the relative levels of *TIMMDC1* transcripts with or without the poison exon (Fig. 7). First, the presence of an 89 bp RT-PCR product (amplified using primers P442/P443 that bind to Exon 3 and Exon 4; the unaltered region of the transcript) showed no aberrant splicing in the mRNA sequence 5′ to the variant and that the *TIMMDC1* mRNA levels were increased in SSO1 and SSO2 compared to the NC5 treated patient fibroblasts (Fig. 7; left panel). Second, the SSO1 and SSO2 treated affected fibroblasts (III-2 and III-6) showed complete disappearance of the 195 bp PCR product (representing the transcripts with the poison exon present in NC5 treated fibroblasts; middle panel, lanes 3, 6) with concomitant appearance of the normally spliced 115 bp RT-PCR product (lanes 1 and 2, 4 and 5 and untreated parent fibroblasts lanes 7 and 8). Third, consistent with the latter data, the levels of 122 bp RT-PCR product—reflecting amplification of *TIMMDC1* mRNA with the poison exon—were reduced in SSO1 and SSO2 treated (right panel; lanes 1 and 2, 4 and 5) compared to NC5 treated (lanes 3, 6) patient fibroblasts. Note that levels of reduction of *TIMMDC1* mRNA with the poison exon in patient fibroblasts approached the levels present in untreated parent fibroblasts (right panel; lanes 7 and 8).

### TIMMDC1 protein levels are reduced in affected LCLs and fibroblasts

**Fig. 8:**
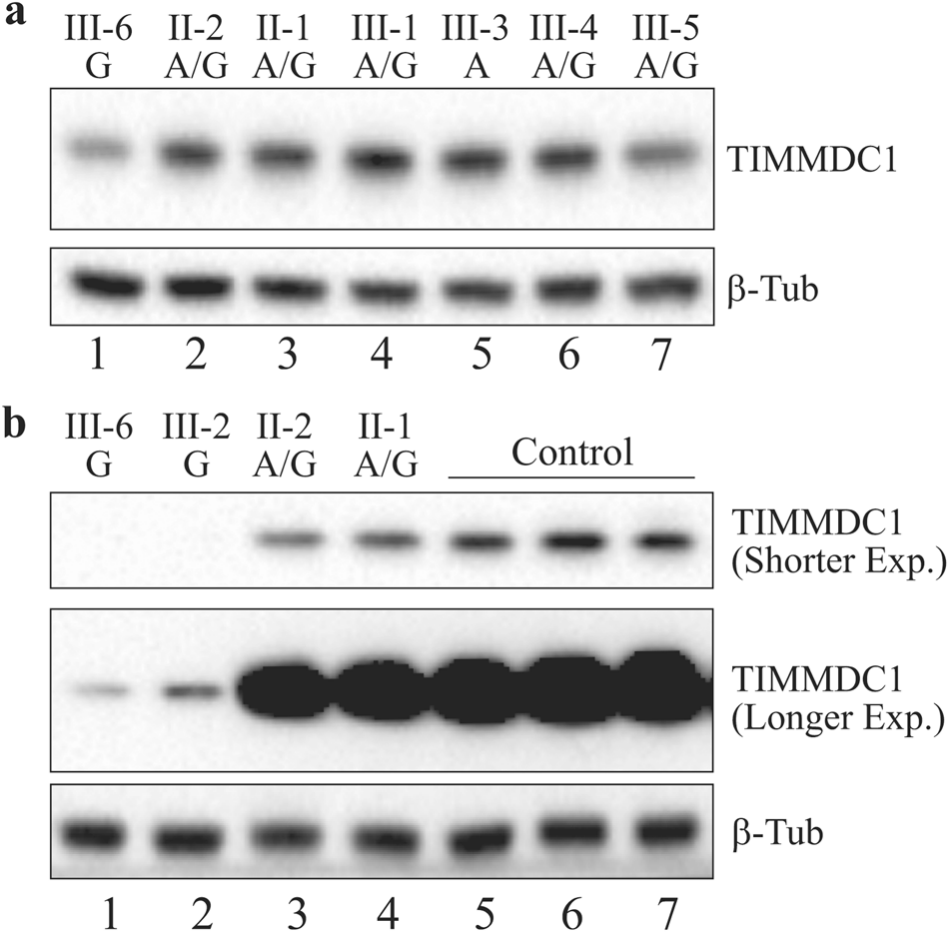
TIMMDC1 protein levels are reduced in the affected LCLs and fibroblasts. **a** Western blot showing moderate reduction in TIMMDC1 protein level in the affected male III-6 LCLs (lane 1) compared to the unaffected family members (lanes 2–7). **b** Western blot showing significant reduction in TIMMDC1 protein level in the affected male (III-6) and female (III-2) fibroblasts (lanes 1, 2) compared to the unaffected carrier parents (II-1 and II-2; lanes 3, 4) and unrelated healthy controls (lanes 5–7). Low levels of TIMMDC1 protein—that was undetectable in shorter exposure—were seen in—the affected (lanes 1, 2) compared to the two carrier-parent (lane 3, 4) fibroblasts. Note that as the parents are heterozygous for the c.597-1340A>G variant, the TIMMDC1 protein level is lower than the unrelated control fibroblasts without this variant. Housekeeping protein β-tubulin was used as a loading control.

The TIMMDC1 poison exon introduces a frameshift resulting in a premature stop codon (p.Gly199_Thr200ins5*) in TIMMDC1 transcripts that are likely degraded via NMD in patient cells (see Fig. 6b). Consequently, the TIMMDC1 protein is undetectable in patient-derived cells^4^. First, we performed western blotting (using antibody against N-terminal 1–100 amino acids) on LCLs available from one affected boy (III-6), the parents and four siblings, and observed reduced levels of TIMMDC1 protein in the affected boy compared to his family members (Fig. 8a, lanes 2–7). We also observed reproducible reduction of the TIMMDC1 protein in one heterozygous sibling (III-5; lane 7). The reason for this is not obvious, but it could be due to unknown cellular changes or cellular selection during the immortalisation of this individual’s B-cells. Second, we determined the TIMMDC1 protein levels in patient, parent, and unrelated control fibroblasts. TIMMDC1 protein levels were negligible in III-6 and III-2 affected fibroblasts (extremely low levels detectable only after very long western blot exposure; Fig. 8b, middle panel, lanes 1, 2) compared to moderately reduced levels in LCLs from III-6 (Fig. 8, lane 1). The low but detectable TIMMDC1 protein in the affected boy’s LCLs (III-6), compared to barely detectable TIMMDC1 protein in his fibroblasts (as seen in the overexposed western blot, Fig. 8b, middle panel) may be due to increased mitochondrial content, higher respiration and proliferation rate (24 h doubling time)^17^, resulting from virus-mediated transformation, as opposed to fibroblasts with lower mitochondrial content (150/cells)^18^ and longer doubling time (47.2 ± 7.5 h)^19^. We also observed reduced TIMMDC1 protein in fibroblasts from the heterozygous parents (Fig. 8b, lanes 3, 4) compared to fibroblasts from unrelated controls without the *TIMMDC1* variant (lanes 5–7).

### TIMMDC1 protein levels are restored in SSO1 and SSO2 treated affected fibroblasts

**Fig. 9:**
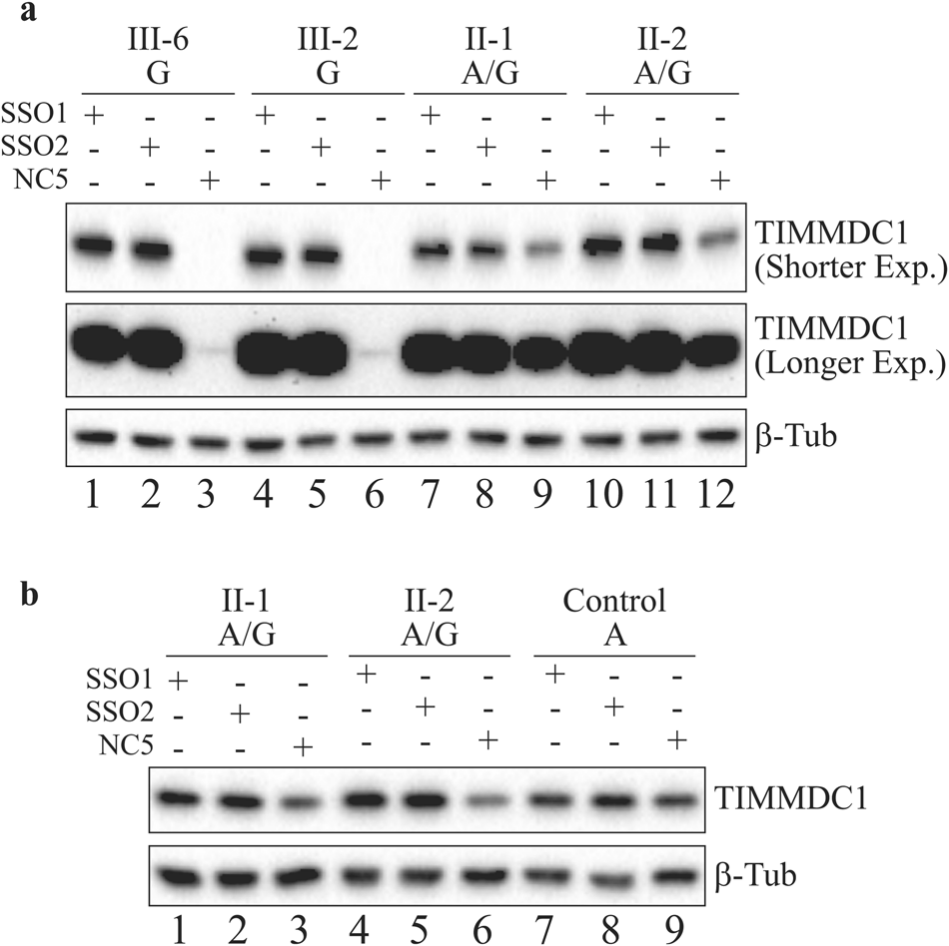
TIMMDC1 protein levels are restored in SSO1 and SSO2 antisense oligonucleotide treated affected fibroblasts. **a** Western blot showing significantly increased TIMMDC1 protein level in SSO1 and SSO2 treated affected (III-2 and III-6; lanes 1, 2 and 4, 5) compared to almost non-existent protein in the NC5 control treated (lanes 3, 6; very low-level signal was detectable after longer exposure) fibroblasts. Note that TIMMDC1 protein level was also increased in SSO1 and SSO2 treated parent (lanes 7, 8 and 10, 11) (heterozygous for the variant) compared to NC5 control (lanes 9 and 12) parent fibroblasts. However, as shown in **b**, no difference in the TIMMDC1 protein levels was observed in SSO1, SSO2 or NC5 control treated healthy unrelated individual’s fibroblasts (lanes 7, 8). Housekeeping protein β-tubulin was used as a loading control.

Having restored normal splicing in the affected fibroblasts (Fig. 7), we determined if TIMMDC1 protein levels also recovered. As expected, TIMMDC1 protein levels recovered in SSO1 and SSO2 treated affected fibroblasts that had negligible basal levels as observed in NC5 treated fibroblasts (Fig. 9a; compare lanes 1, 2 with 3 and 4, 5 with 6 in longer exposure). Interestingly, TIMMDC1 protein levels also increased in the SSO1 and SSO2 but not NC5 treated heterozygous parent fibroblasts (Fig. 9a; top panel, compare lanes 7, 8 with 9 and 10, 11 with 12). We also compared the levels of TIMMDC1 protein recovery in SSO1 and SSO2 treated parent fibroblasts (heterozygous for the variant) with the homozygous healthy control without the variant fibroblasts. As expected, there was a nearly two-fold increase in TIMMDC1 protein levels in SSO1 and SSO2 compared to NC5 treated parent fibroblasts (Fig. 9b; compare lanes 1, 2 with 3 and 4, 5 with 6). However, no such increase was observed in SSO1 and SSO2, compared to NC5 treated, healthy control fibroblasts (Fig. 9; compare lanes 7, 8 with 9).

### Mitochondrial complex I proteome is restored in SSO1 treated affected fibroblasts

**Fig. 10:**
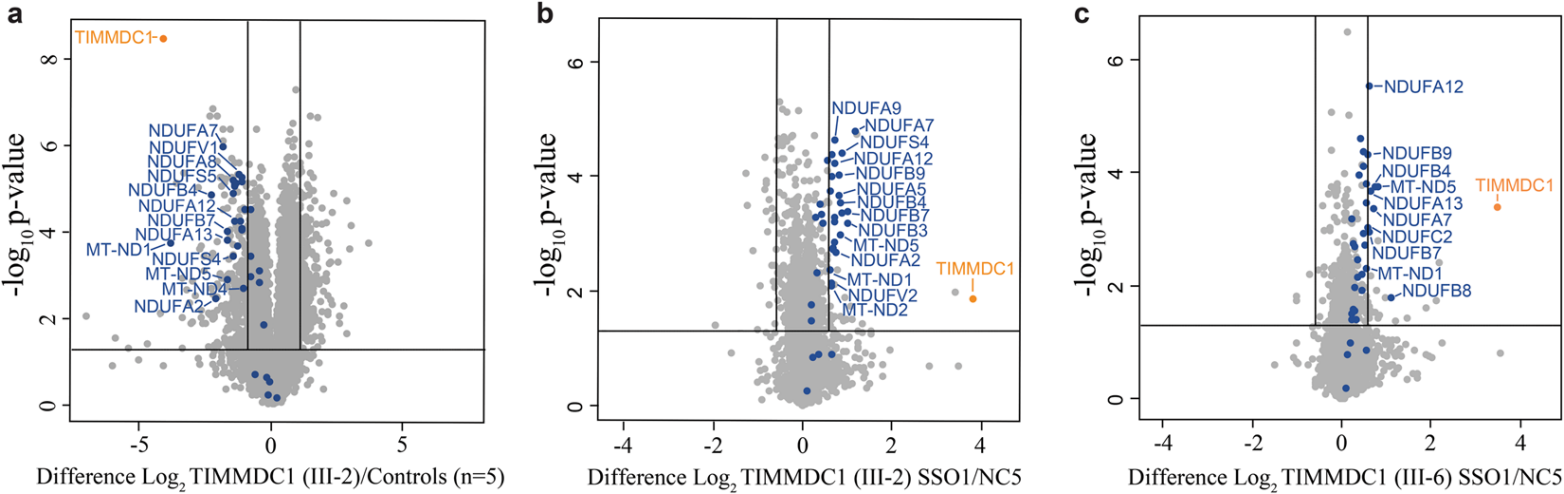
Mitochondrial complex I proteome is restored in SSO1 treated affected fibroblasts. **a** Volcano plot expressing quantitative proteomics data showing a decreased abundance of TIMMDC1 and other complex I subunits in patient III-2 relative to healthy control (*n* = 5) whole fibroblasts. **b** Volcano plot showing increased abundance of TIMMDC1 and other complex I subunits in SSO1 relative to NC5 treated patient III-2 or **c** III-6 whole fibroblasts. Horizontal line within the volcano plots represents a significance of *p* = 0.05 and vertical lines represent a fold-change of ±1.5. Blue = complex I subunits.

TIMMDC1 deficiency has been shown to affect levels of other complex I subunits and consequently significantly reduces mitochondrial function^9,20,21^. TIMMDC1 is implicated in the biogenesis of the ND1 subunit of complex I, one of the 13 proteins encoded by mitochondrial DNA^7–9^. In line with this, CRISPR/Cas9 knockout of *TIMMDC1* in the human embryonic kidney 293 cell line leads to a severe reduction in the relative abundance of ND1^7,22^ and complex I subunits directly associated with ND1 (subunits in the so-called ND1 module), as well as a general reduction in the relative abundance of other complex I subunits^22^. We performed label-free (data-independent acquisition) based quantitative proteomics on *TIMMDC1* patients (III-2) and control fibroblasts (Fig. 10a; Supplementary Data 1). Similar to what was observed in *TIMMDC1* knockout cells^22^, III-2 fibroblasts have significantly lower levels of ND1 (*MT-ND1*) compared to controls and concomitant reduction in the levels of ND1 module subunits NDUFA8 and NDUFA13 as well as other complex I subunits (Fig. 10a, complex I subunits labelled in blue). We next performed quantitative proteomics on fibroblasts from both affected homozygous siblings treated with either SSO1 or NC5, finding SSO1 treatment to efficiently increase the relative levels of TIMMDC1 protein itself, ND1 and other complex I subunits (Fig. 10b, c).

### Mitochondrial function is restored in SSOs treated patient fibroblasts

**Fig. 11:**
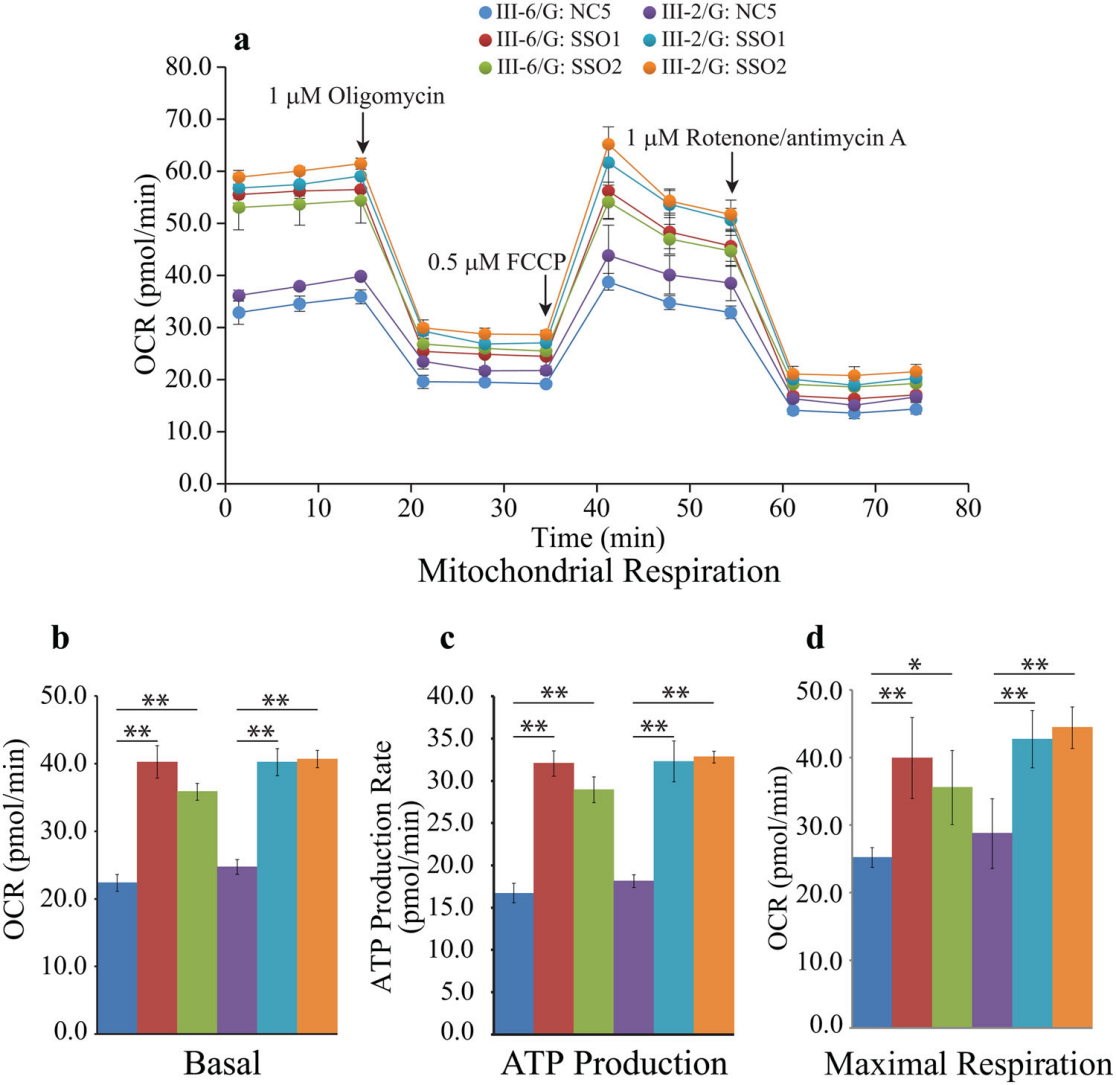
Mitochondrial function is restored in SSO treated affected fibroblasts. **a** Oxygen consumption rate (pmol/min) at baseline conditions and then after oligomycin, FCCP and rotenone/antimycin A injections. **b** Basal respiration, **c** ATP production, and **d** Maximal respiration were measured in SSO1, SSO2 or NC5 treated patient fibroblasts. Results are expressed as mean ± SEM. *p < 0.05; **p < 0.01 (two-tail Student’s *t* test).

Due to severe reduction in the abundance of TIMMDC1 protein and complex I subunits in the patient fibroblasts relative to controls, we predicted this would result in a substantial loss of mitochondrial function, particularly ATP production. As we could restore normal TIMMDC1 splicing, protein levels, and the levels of complex I subunits in SSO treated patient fibroblasts, we explored if SSO treatment also restored mitochondrial function. We measured oxygen consumption rate (OCR; pmol/min), ATP production and maximal respiration at baseline conditions and then after oligomycin, FCCP and rotenone/antimycin A injections in SSO1, SSO2 or NC5 treated patient fibroblasts (Fig. 11). Patient fibroblasts showed low basal levels of OCR, ATP production and maximal respiration that were significantly increased on SSO1 or SSO2, but not NC5, treatment (Fig. 11). However, no change in these parameters was observed in SSO1, SSO2 treated skin fibroblasts from either carrier parent (Supplementary Fig. 2).

## Discussion

Many intronic variants causing neurological disorders have been identified using RNA analysis of patient-derived cells or tissues, after initial efforts to identify a causative variant via gene panel sequencing, whole exome and genome sequencing have failed (about 50% of cases)^23,24^. This has prompted the use of RNAseq along with genome sequencing data to identify disease variants in unresolved cases and it is now clear that variants causing aberrant splicing and/or gene expression are not restricted to splice-site junctions and are also present in intronic or intergenic sequences^25^. Unfortunately, RNAseq analysis of easily accessible tissues (e.g., fibroblasts, blood, or muscle) will still run short of solving many neurological disorders and disease-relevant tissues will be required, e.g., neurons derived from induced pluripotent stem cells generated by reprogramming of patient fibroblasts or lymphoblasts.

The siblings we describe here were reported previously by us^5^ to have a homozygous variant in *STXBP5L* (c.3127G>A, p.Val1043Ile) associated with a phenotype summarised as an infantile-onset neurodegenerative disorder, manifesting a predominant sensorimotor axonal neuropathy, optic atrophy and cognitive deficit. The STXBP5L p.Val1043Ile variant proved highly conserved but was otherwise predicted to have minimal impact on protein function; however, subsequent functional evidence suggested consideration towards a pathobiological consequence of this *STXBP5L* gain of function variant^5^. Furthermore, while *Stxbp5l* null mice demonstrate a neuromuscular phenotype^26^, supportive of this hypothesis, a conclusive explanation for the observed human phenotype remained elusive. Therefore, the contribution of the STXBP5L p.Val1043Ile variant to the clinical manifestations of our affected sibs remains unclear.

A subsequent report by Kremer et al. presented three unrelated children with a homozygous deep intronic *TIMMDC1* variant (c.596+2146A>G, now annotated as c.597-1340A>G), whose clinical features we considered similar to those of the siblings we reported in 2015^4,5^ (see Supplementary Table 1). A further report of the *TIMMDC1* c.597-1340A>G variant by Naber et al.^10^ described compound heterozygosity for the *TIMMDC1* c.597-1340A>G and c.385C>T variants, in siblings with a comparable, though more severe phenotype and death secondary to respiratory insufficiency within the first year of life^10^. The clinical features of the seven published cases with the *TIMMDC1* c.597-1340A>G variant are presented in Supplementary Table 1).

Considering *TIMMDC1* maps to the identity by descent region of our family, we reanalysed the RNAseq data from our previously published siblings^5^ and identified the same *TIMMDC1* c.597-1340A>G variant in our patients. Subsequent molecular and cellular functional studies supported pathogenicity resulting from loss of TIMMDC1, an assembly factor critical to biogenesis of complex I of the mitochondrial ETC^9^. Complex I (nicotinamide adenine dinucleotide (NADH):ubiquinone oxidoreductase) is a multimeric complex comprising 45 subunits (synthesised from 44 different genes) and the first enzyme of the mitochondrial ETC critical to oxidative phosphorylation responsible for ATP synthesis. Resulting from impaired ETC, primary mitochondrial diseases are clinically heterogeneous and can be caused by variants in more than 300 genes encoded by either nuclear or mitochondrial DNA^27^; however, in childhood neurological presentations, variants in nuclear genes predominate^28^, one of which is the mitochondrial inner membrane protein, TIMMDC1^9,22^.

As is typical of most mitochondrial disorders, nuclear complex I disease manifests a range of overlapping phenotypes. Spanning characteristic syndromic presentations such as fatal infantile lactic acidosis, Leber’s hereditary optic neuropathy and most frequently, subacute necrotising encephalomyelopathy of Leigh syndrome spectrum, to more variable phenotypes, the majority of which present with the unremitting neurological decline within the first year of life^11^. While the small number of cases reported precludes the assertion of a clear genotype–phenotype relationship, it is notable that the two cases demonstrating early infantile death were compound heterozygous for the *TIMMDC1* exonic c.385C>T and the deep intronic c.597-1340A>G variants^10^; this raises the possibility of a survival benefit conferred by the small proportion of properly spliced *TIMMDC1* transcripts that are present in the context of this intronic variant.

Our RT-PCR amplification of poison exon-specific *TIMMDC1* transcripts showed the presence of higher levels of this mRNA in patient fibroblasts compared to very low levels in the homozygous healthy individual (see lane 5, P446-P448 PCRs; Fig. 6a) suggesting that the *TIMMDC1* c.597-1340A>G variant indeed enhances insertion of an underutilized (poison) exon into the *TIMMDC1* mRNAs in patient fibroblasts. However, OCR levels (Fig. 11) in patient fibroblasts suggested that a low level of normal *TIMMDC1* transcript (P444/P448 PCR; Fig. 6a), and thus the translated protein (Fig. 8b), is sufficient for minimal mitochondrial function and thus enough for survival, albeit at the expense of severe, and ultimately fatal disorder. The carrier parents showed almost half the poison *TIMMDC1* mRNA levels (compared to the two affected children, lanes 3, 4, P446–P448 PCRs; Fig. 6a), consistent with the observation that the unaffected parents have half the TIMMDC1 protein (compared to healthy controls; Fig. 8b). That homozygous *Timmdc1* null mice are embryonically lethal (see: https://www.mousephenotype.org/data/genes/MGI:1922139#diseases) further underscores the importance of TIMMDC1 protein for cell function.

Lake et al. concluded that the p.Arg225* *TIMMDC1* nonsense variant was hypomorphic and an unlikely contributor to pathogenesis in a patient with Leigh syndrome, given that the presence of truncated TIMMDC1 protein had almost no effect on mitochondrial complex I assembly and activity in patient fibroblasts homozygous for the variant^21^. However, homozygosity for the c.847delG (p.Asp283fs+54 aa) *TIMMDC1* variant in a patient with Leigh syndrome was shown to have barely detectable TIMMDC1 protein, and thus lower levels of ATP content, mitochondrial respiratory activity and complex I in patient LCLs compared to control cells^20^. In contrast to the p.Arg225* truncated but stable TIMMDC1 protein^21^, and the p.Asp283fs+54 aa recombinant but unstable TIMMDC1 protein^20^, we could not detect a truncated p.Gly199_Thr200ins5* TIMMDC1 protein (even in the presence of proteasomal inhibitor MG132; *data not shown*). However, we could detect extremely low levels of full-length protein in our patient fibroblasts (e.g., longer exposures in Figs. 8b, 9a). Whereas the p.Arg225* and p.Asp283fs+54 aa TIMMDC1 variants have mild to moderate impact on complex I assembly, consistent with the consequence of *TIMMDC1* knockout^20,21^, complex I assembly is completely abolished in *TIMMDC1* p.Gly199_Thr200ins5* fibroblasts^4^. More recently, a mitochondrial gene panel was used to identify a heterozygous pathogenic *TIMMDC1* c.385C>T p.Arg129* variant, and targeted Sanger sequencing a heterozygous deep intronic *TIMMDC1* c.597-1340A>G; p.Gly199_Thr200ins5* variant, in two affected brothers; both were compound heterozygous for the two *TIMMDC1* pathogenic variants and, consistent with autosomal recessive inheritance, their unaffected parents were heterozygous for one of the two variants^10^. Taken together, these observations are consistent with a low level of mitochondrial function in our patients’ fibroblasts (Fig. 11) that is sufficient for cell proliferation.

TIMMDC1 is a critical assembly factor for mitochondrial complex I. In Drosophila, *TIMMDC1* is essential for viability and zebrafish *timmdc1* is required for normal behaviour and neurological function^20^. In cultured human cells, TIMMDC1 depletion significantly reduces the activity of mitochondrial complex I but not complexes II-IV^29^, while total ablation of expression with CRISPR/Cas9 leads to specific loss of ND1 translation^7^. ND1 is one of the 13 protein-coding genes present on mitochondrial DNA and the core subunit for the so-called ND1 module, a domain of complex I that sits at the intersection between the proton-pumping membrane arm of the complex and the catalytic NADH dehydrogenase domain protruding into the mitochondrial matrix. The absence of all nuclear-encoded subunits present in the ND1 module leads to severely impaired complex I assembly, loss of specific complex I enzyme activity and mitochondrial respiration^22^. Consistent with its role in ND1 biogenesis, TIMMDC1 knockout or knockdown leads to turnover of ND1 module and other complex I subunits, reduced levels or absence of mature complex I, and a reduction in cellular oxygen consumption rate^8,9,20^. In c.597-1340A>G; p.Gly199_Thr200ins5* patient fibroblasts, TIMMDC1 is undetectable and complex I assembly is significantly reduced, but its re-expression restores complex I to normal levels^4^. In longer exposures of western blots, we detected extremely low levels of TIMMDC1 protein in fibroblasts from our two patients (Fig. 8b), which also exhibited low levels of basal mitochondrial respiration (Fig. 11). These results, and the higher levels of TIMMDC1 observed in LCLs (Fig. 8a), imply that these patients express levels of TIMMDC1 sufficient for the proliferation of some cell types, but not of those key cell types whose abnormal function determines patient phenotype, for example, neural and muscle cells.

There is an unmet need for targeted therapies for mitochondrial disease and here, for the first time, we demonstrate that two SSOs targeting the *TIMMDC1* c.597-1340A>G variant can restore mitochondrial function in patient cells, suggesting that the functional effect of the aberrant splicing can be repaired. Indeed, the near-complete disappearance of poison exon *TIMMDC1* transcripts and concomitant restoration of TIMMDC1 protein levels (in fact, to above parental levels) in the SSO1 and SSO2, but not NC5, treated fibroblasts suggests successful suppression of aberrant splicing (Figs. 7 and 9). Compared with NC5, the SSO1 and SSO2 treatment showed an almost two-fold TIMMDC1 protein increase in heterozygous parental but not in homozygous healthy control fibroblasts (Fig. 9b), which is consistent with suppression of aberrant splicing of mRNAs transcribed from the *TIMMDC1* variant allele. We also observed restoration of complex I subunit levels (Fig. 10), OCR, ATP production and respiration concomitant with increased levels of TIMMDC1 protein in SSO1 and SSO2, but not NC5, treated variant fibroblasts (Figs. 9 and 11).

There have been rapid improvements to the structure and chemistry of SSOs that have led to their clinical use, e.g., for treating spinal muscular atrophy, Duchenne muscular dystrophy and amyotrophic lateral sclerosis (reviewed in refs. ^30,31^). Overall, 10 oligonucleotide drugs, six of which are SSOs, have been approved by FDA^32,33^ and others are in late stages of clinical trials, e.g., Sepofarsen targeting a commonly occurring deep intronic *CEP290* variant that causes Leber congenital amaurosis^34^. Together with FDA approved Milasen, a personalised SSO that silences a cryptic splice site introduced by insertion of a retrotransposon in *CLN7* gene, causing a rare, fatal neurodegenerative Batten’s disease in a single patient^35^, our findings support further studies to assess the potential of SSOs for treating patients with the *TIMMDC1* c.597-1340A>G variant, and cognate variants.

Patients with the intronic *TIMMDC1* variant described here are estimated to have a prevalence of 1/25 million in the European population, based on the gnomAD c.597-1340A>G allele frequency of 0.0001946. Besides emphasising the importance of improved transcriptomic-based genetic diagnosis, our data provide insights into the mechanisms of mitochondrial dysfunction—both molecular and cellular—caused by the *TIMMDC1* c.597-1340A>G variant. That two SSOs targeting the TIMMDC1 c.597-1340A>G variant can restore mitochondrial function in patient cells is encouraging and raises the possibility of developing targeted treatment, where none currently exists. Therefore, although not without challenges due to differences in splicing factors and divergent exon 5 and 6 intronic sequences between human and mouse, generating a *TIMMDC1* c.597-1340A>G humanised preclinical mouse model that recapitulates the human disease phenotype, in comparison with patient-derived iPSC models, will be a logical next step. Nonetheless, both approaches will facilitate an in-depth understanding of the molecular and cellular mechanisms of the disease, and provide opportunities for assessing the potential of SSOs as therapeutics for TIMMDC1 and other disorders with similar genetic mechanisms.

## Methods

### Ethics statement

The study was approved by the Women’s and Children’s Health Network Human Research Ethics Committee (HREC 2361/3/2023) and informed written consents were obtained for all individuals on whom genetic testing and/or molecular investigations were performed.

### Cell culture—fibroblast and LCL maintenance and transfections

Lymphoblastoid cell lines (LCLs) from all healthy family members and one affected family member (III-6) were established by infecting peripheral blood lymphocytes with Epstein-Barr Virus using a published method^36^. Established LCLs were maintained in RPMI-1640 (Sigma-Aldrich) supplemented with 10% foetal bovine serum (Thermo Fisher Scientific), 2 mM l-glutamine (Sigma-Aldrich), and 0.15 mg/ml benzylpenicillin (CSL Limited, Melbourne, Australia) at 37 °C with 5% CO_2_. Fibroblasts derived from the parents and two affected children were maintained in Dulbecco’s Modified Eagle’s medium (Sigma-Aldrich) containing 2 mM l-glutamine, 1 mM sodium pyruvate, and 10% foetal bovine serum at 37 °C with 5% CO_2_.

For reverse transcription-polymerase chain reaction (RT-PCR) and western blot analysis, fibroblasts (10^5^/well) were plated in six-well dishes. Next day, the cells were transfected with 100 nM *TIMMDC1* SSO1, SSO2 or NC5 (non-specific control) splice-switching oligonucleotides (SSOs) (IDT) using Lipofectamine RNAiMAX (ThermoFisher) following vendor’s protocol and harvested 48 h later.

### Blood DNA PCR and Sanger sequencing

The genomic region flanking the c.597-1340A>G *TIMMDC1* variant was amplified from blood DNA of all family members using Platinum SuperFi PCR Master Mix (Thermo Fisher) and Primers P445/P448 (Supplementary Table 3) under the following conditions: 95 °C for 2 min; 36 cycles of 95 °C for 15 s, 59 °C for 15 s, 68 °C for 2 min and final one cycle of 68 °C for 10 min, PCR products gel-purified and subjected to Sanger sequencing.

### RNA extraction and RT-PCR

RNA was isolated from SSO1, SSO2 or NC5 treated fibroblasts using RNeasy Mini Kit and on-column RNase-free DNase-treatment (Qiagen). cDNAs were generated from the total RNA (500 ng each) using Superscript IV reverse transcriptase (Thermo Fisher) and subjected to PCR amplification using different pairs of primers (Fig. 2; Supplementary Table 3). PCRs were performed using Phusion High-Fidelity DNA Polymerase (NEB) under the following conditions: 98 °C for 30 s; 31 cycles of 98 °C for 10 s, 58 °C for 10 s, 72 °C for 15 s and final one cycle of 72 °C for 10 min. Some of the PCR products were gel purified and subjected to Sanger sequencing.

### Genome sequencing

Mapping of read data to the 1000 genomes project build 37 of the human genome (available from http://ftp.1000genomes.ebi.ac.uk/vol1/ftp/technical/reference/human_g1k_v37.fasta.gz) was performed as described as part of our previous study^5^. Variants located within a region starting 3.5 kb upstream of *TIMMDC1* and concluding at the end of the final exon according to the NM_016589.4 reference sequence (GRCh37 NC_000003.11:119213926_119243175) were called using bcftools mpileup and bcftools call v1.9 with default settings and the resulting variant call format (VCF) file was annotated using ANNOVAR^37^, SpliceRover^38^, DSSP^39^ and SpliceAI^40^. Read alignments were viewed with the Integrative Genomics Viewer (IGV) v.2.5.2.

### RNAseq

RNA was extracted from fibroblasts derived from individuals with neurodevelopmental disorders (*n* = 171) or their unaffected family members (*n* = 107) as controls, this included parents (II-1, II-2) and patients (III-2, III-6) from our study. cDNA libraries were prepared using the TruSeq Stranded mRNA protocol (Illumina). Reads were mapped to the GRCh38 build of the human genome using STAR v2.7.3a^41^. Read counts were generated using FeatureCounts v1.6.3 and detection of outliers, based on false discovery rate (FDR) of <0.05, was performed using edgeR v3.24.3. Detection of novel outlier splicing events was performed using LeafCutter v0.2.9^42^ with significant alternative introns defined by a FDR < 0.05. Sashimi plots showing splicing patterns were generated using IGV.

### Western blot assays

Fibroblasts were lysed in 50 mM Tris–HCl pH 7.5, 250 mM NaCl, 0.1% Triton-X-100, 1 mM EDTA, 50 mM NaF, 0.1 mM Na_3_VO_4_, 1× Protease inhibitor/No EDTA and LCLs in 50 mM Tris–HCl pH 7.5, 50 mM KCl, 0.1% NP40, 5 mM EDTA, 50 mM NaF, 0.1 mM Na_3_VO_4_, 1× Protease inhibitor/No EDTA^43^, sonicated with microtip (10 s 25% amplitude; Sonics Vibra-Cell VCX 130), cell debris removed by centrifugation at 15,000 × *g* for 20 min at 4 °C and proteins assayed using BCA assay kit (ThermoFisher). 8 µg protein of each sample was resolved on 4–12% Bis–Tris protein gels (ThermoFisher), transferred to nitrocellulose membranes and western blotted with rabbit monoclonal anti-TIMMDC1 antibody (1:2000; ab171978; Abcam) or anti-β-Tubulin (1:5000; Ab6046; Abcam) and polyclonal goat anti-rabbit IgG/HRP (1:1000; P0448; Dako) as secondary antibody. Signal was detected by Clarity Western ECL Substrate (Bio-Rad) and captured using Gel Documentation System (Bio-Rad). All blots and gels shown are derived from the same experiments and were processed in parallel.

### Quantitative mass spectrometry and data analysis

For whole fibroblast quantitative proteome analyses, 700,000 fibroblasts/T75 flask (in triplicate for each treatment) of patients III-2, III-6 and controls were plated in 15.6 ml Dulbecco’s Modified Eagle’s medium (Sigma-Aldrich) containing 2 mM l-glutamine, 1 mM sodium pyruvate, and 10% foetal bovine serum at 37 °C with 5% CO_2_. Next day, III-2 and III-6 fibroblasts were transfected with 100 nM TIMMDC1 SSO1 or NC5 (non-specific control) using Lipofectamine RNAiMAX (Thermo Fisher) following vendor’s protocol, harvested 48 h later and snap-frozen for subsequent proteome analysis. Fibroblasts cell pellets were solubilised in 5% SDS, 50 mM Triethylammonium bicarbonate and total protein quantified using Pierce BCA protein assay kit (Thermo Fischer). A total of 25 µg protein from two experiments: (1) untreated III-2 fibroblasts (in triplicate and 5 healthy controls in singlicate) and (2) III-2 or III-6 SSO1 or NC5 treated fibroblasts (all in triplicate) were processed using S-trap micro spin columns per manufacturer’s instructions (ProtiFi). Proteins were digested at 1:10 trypsin to protein ratio at 37 °C overnight and peptides were eluted over three elution steps as per manufacturer’s instructions (ProtiFi). Samples were dried down using a CentriVap Benchtop Vacuum Concentrator (Labconco), reconstituted in 45 µL of 2% acetonitrile, 0.1% trifluoroacetic acid buffer and 2 µL were injected for liquid chromatography (LC)-tandem mass spectrometry (MS/MS). The LC system was equipped with an Acclaim Pepmap nano-trap column (Dinoex-C18, 100 Å, 75 µm × 2 cm) and an Acclaim Pepmap RSLC analytical column (Dinoex-C18, 100 Å, 75 µm × 50 cm). The tryptic peptides were injected to the enrichment column at an isocratic flow of 5 µL/min of 2% v/v acetonitrile containing 0.1% v/v formic acid for 5 min applied before the enrichment column was switched in line with the analytical column. The eluents were 5% dimethyl sulphoxide in 0.1% v/v formic acid (solvent A) and 5% dimethyl sulphoxide in 100% v/v acetonitrile and 0.1% v/v formic acid (solvent B). The flow gradient was (i) 0–6 min at 3% B, (ii) 6–7 min, 3–4% (iii) 7–82 min, 4–25% B (iv) 82–86 min, 25–40% B (v) 86–87 min, 40–80% B (vi) 87–90 min, 80–80% B (vi) 90–91 min, 80–3% and equilibrated at 3% B for 10 min before the next sample injection. The mass spectrometer was operated with a data-independent acquisition method. Briefly, full MS resolutions were set to 120,000 at *m*/*z* 200 and scanning from 350 to 1400*m*/*z* in the profile mode. Full MS AGC target was 250% with an IT of 50 ms. AGC target value for fragment spectra was set at 2000%. 50 windows of 13.7 Da were used with an overlap of 1 Da. Resolution was set to 30,000 and maximum IT to 55 ms. Normalised collision energy was set at 30%. All data were acquired in centroid mode using positive polarity. Raw files were processed with Spectronaut (v.115.2.210819.50606, Rubin) against a data-dependent acquisition derived library containing 124,887 precursors from deeply fractionated control fibroblast samples. Default BSG Factory search parameters were used with a few modifications. “Exclude single hit proteins” option was selected, “Major Group Top N” and “Minor Group Top N” was unselected to allow all peptides to be considered for quantitation, “Data filtering option” was set to “*Q*-value sparse” and “Imputing strategy” was set to “no imputing”. Protein search was done using UniProt reviewed human canonical and isoform (42,386 entries). Data processing and statistical analysis were performed in Perseus (v.1.6.14.0)^44^. MS2 quantities were log2-transformed and samples were labelled into their respective groups. Samples were normalised by subtracting the mean of all data points for each sample. MitoCarta 3.0 database was used to annotate the entries using the UniProt IDs^45^. Groups were filtered to have at least two valid values for the two-sample *t*-test. Volcano plots were generated using scatter-plot function in Perseus and significance lines were set to *p*-value = 0.05 (−log_10_ = 1.301) and fold-change ± 1.5 (log_2_ ±0.585).

### Mito stress assay

Mitochondrial function was assayed by measuring the oxygen consumption rate (OCR) in adherent fibroblasts with Seahorse XF96 Extracellular Flux Analyser kit (Agilent Technologies). Fibroblasts (six wells for each treatment) were seeded in 96-well culture microplate (Seahorse Technologies) pre-treated overnight with 5 µg/ml fibronectin (Sigma-Aldrich) at 10,000 cells/well in 100 µl DMEM (D5671; Sigma-Aldrich) with 1 mM sodium pyruvate, 2 mM l-glutamine and 10% FCS, and incubated for 24 h at 37 °C in 5% CO_2_. Next day, the cells were transfected with 100 nM TIMMDC1 SSO1, SSO2 or NC5 antisense oligonucleotides using Lipofectamine RNAiMAX (ThermoFisher) following vendor’s protocol. Forty-eight hours later, the growth medium was replaced with 180 µl bicarbonate-free pre-warmed Seahorse XF DMEM medium pH 7.4 supplemented with 1 mM sodium pyruvate, 2 mM l-glutamine and 10 mM glucose, and incubated at 37 °C for 40 min in CO_2_-free incubator before starting the assay. All assay procedures were as recommended for the Agilent Seahorse XF cell mito stress kit. The basal respiration rate and the data from cell sequentially treated with 1 µM oligomycin (ATP synthase inhibitor), 0.5 µM FCCP (electron transport uncoupler) and 1 µM rotenone/antimycin A (complex I and complex III inhibitors, respectively) were collected. The data was analysed using XF Wave software according to manufacturer’s instructions.

### Reporting summary

Further information on research design is available in the Nature Research Reporting Summary linked to this article.

## Supplementary information

Supplementary material.

Reporting Summary

Supplementary Data 1

## Data Availability

In accordance with informed consent signed by the participants, sharing of supporting details is subject to ethical review by the Adelaide Women’s and Children’s Health Network (WCHN) Human Research Ethics Committee. Requests for data can be made by contacting the corresponding author (J.G.). The mass spectrometry proteomics data have been deposited to the ProteomeXchange Consortium via the PRIDE (http://www.ebi.ac.uk/pride) partner repository with the dataset identifier PXD029707. Proteomics data has also been provided as Supplementary Data 1.
