# Supplementary material for: Oligonucleotide correction of an intronic TIMMDC1 variant in cells of patients with severe neurodegenerative disorder
Source: NPJ Genom Med. 2022 Jan 28;7:9. doi: 10.1038/s41525-021-00277-7 (PMC8799713; doi:10.1038/s41525-021-00277-7)
Supplement: Supplementary file 1 — Supplementary material. [file 41525_2021_277_MOESM1_ESM.pdf]

Reference Sequence

agTTGGT**A**TTTGT***CTGACTA***GAAGAGTGCTACCTTCGTCCTCCACCTGTTCACCCCTTTGTCTACCTCCTTTGAAGGAGAAGgt

Variant Sequence

agTTGGT**G**TTTGT***CTGACTA***GAAGAGTGCTACCTTCGTCCTCCACCTGTTCACCCCTTTGTCTACCTCCTTTGAAGGAGAAGgt

c.597-1340A>G   Exonic Splicing Enhancer

Supplementary Fig. 1 The reference and c.597-1340A>G 80 bp poison exon sequences (uppercase) are shown with the position of the variant indicated in bold and underlined. The expected binding site for SRSF1 predicted by ESEfinder is indicated in blue italics. SRSF2 and SRSF5 were also predicted by ESEfinder to bind to motifs overlapping the SRSF1 site.

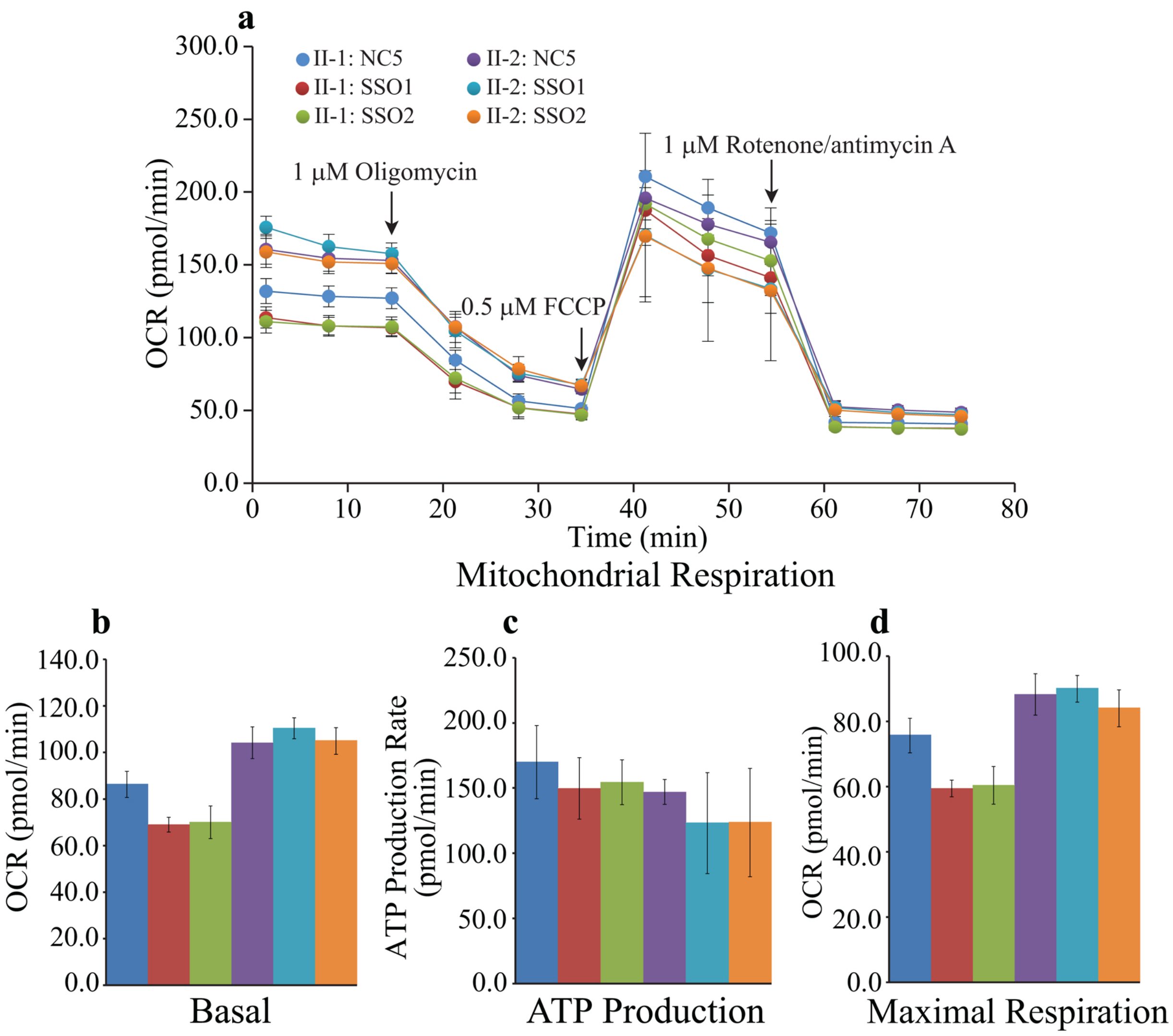

Supplementary Fig. 2 Mitochondrial function is unaffected in SSO treated parent fibroblasts. Oxygen consumption rate (pmol/min) at baseline conditions and then after oligomycin, FCCP and rotenone/antimycin A injections was measured in SSO1, SSO2 or NC5 treated parent fibroblasts seeded at 50,000 cells/well of 96 well

Supplementary Table 1 *TIMMDC1*-Related Disease: Clinical Features

	Naber et al. (2021)	Naber et al. (2021)	Kremer et al. (2017)	Kremer et al. (2017)	Kremer et al. (2017)	Kumar et al. (2015)	Kumar et al. (2015)
**Case identifier**	**II-1**	**II-3**	**Case 1 #35791**	**Case 2 #66744**	**Case 3 #96687**	**III-2**	**III-6**
***TIMMDC1* variant**	c.385C > T, p.(Arg129*)<br><br>596 + 2146A > G, p.(Gly199_Thr200ins5*)	c.385C > T, p.(Arg129*)<br><br>596 + 2146A > G, p.(Gly199_Thr200ins5*)	c.596 + 2146A > G, p.(Gly199_Thr200ins5*)<br><br>c.596 + 2146A > G, p.(Gly199_Thr200ins5*)	596 + 2146A > G, p.(Gly199_Thr200ins5*)<br><br>c.596 + 2146A > G, p.(Gly199_Thr200ins5*)	c.596 + 2146A > G, p.(Gly199_Thr200ins5*)<br><br>c.596 + 2146A > G, p.(Gly199_Thr200ins5*)	c.596 + 2146A > G, p.(Gly199_Thr200ins5*)<br><br>c.596 + 2146A > G, p.(Gly199_Thr200ins5*)	c.596 + 2146A > G, p.(Gly199_Thr200ins5*)<br><br>c.596 + 2146A > G, p.(Gly199_Thr200ins5*)
**Sex**	Male	Male	Male	Male	Male	Female	Male
**Ancestry**	Dutch	Dutch	Greek	North African	German	Middle Eastern	Middle Eastern
**Age at presentation (months)**	2.5 m	1 m	< 12 m	6 m	3 m	≤ 0.5 m	4 m
**Age at death (years, months)**	6.5 m	3.5 m	2 y, 6 m	1yr, 8 m	Alive at 4y[(1)]	2 y, 6 m	7 y, 8 m
**Presenting features**	**Infantile onset:**<br><br>Hypotonia<br><br>Feeding difficulties (nasogastric feeding required)<br><br>Failure to thrive<br><br>Respiratory insufficiency (mechanical ventilation required)	**Infantile onset:**<br><br>Hypotonia<br><br>Feeding difficulties (nasogastric feeding required)<br><br>Failure to thrive<br><br>Respiratory insufficiency (mechanical ventilation required)	**Infantile onset:**<br><br>Hypotonia<br><br>Feeding difficulties<br><br>Failure to thrive<br><br>Developmental delay	**Infantile onset:**<br><br>Hypotonia<br><br>Developmental delay<br><br>Nystagmus	**Infantile onset:**<br><br>Hypotonia<br><br>Feeding difficulties<br><br>Failure to thrive	**Infantile onset:**<br><br>Hypotonia<br><br>Feeding difficulties<br><br>Failure to thrive<br><br>Irritability	**Infantile onset:**<br><br>Hypotonia<br><br>Feeding difficulties (mild)
**Neurological features[(2)]**							
**Hypotonia**	✓<br>(Axial + Appendicular)	✓<br>(Axial + Appendicular)	✓<br>(pattern NR)	✓<br>(pattern NR)	✓<br>(pattern NR)	✓<br>(Axial + Appendicular)	✓<br>(Axial + Appendicular)
**Weakness / muscle wasting**	✓	✓	✓	NR	✓	✓	✓
**Reduced / absent myotatic reflexes / Peripheral neuropathy**	✓	✓	NR	✓	(✓)	✓	✓
**Dysautonomia[(3)]**	✓	✓	NR	NR	NR	(✗)	(✗)
**Motor delay / regression**	✓	✓	✓	(✓)	✓	✓	✓
**Extrapyramidal**	NR	NR	✓<br>(Dyskinesia)	NR	NR	✓<br>(Dystonia + Dyskinesia)	(✗)

**Cognitive delay / regression**	NR	NR	✓	✓	✓	✓	✓
**Epilepsy**	✗<br>-Apnea of undefined etiology	✗<br>-Apnea of undefined etiology	NR	✓<br>-Single presumed seizure with myoclonus and abnormal ocular movements	✓<br>-Pharmacoresistant	✓<br>-Pharmacoresistant<br>-Generalized Tonic-Clonic<br>-Myoclonic<br>-Convulsive Status Epilepticus	✓<br>-Pharmacoresistant<br>-Generalized Tonic-Clonic<br>-Epilepsia partialis continua
**Ophthalmoparesis**	✓	NR	NR	NR	NR	✓<br>-Lateral progressing to complete	✓<br>-Intermittent esotropia progressing to complete
**Optic atrophy**	NR	NR	NR	NR	NR	✓	✓
**Nystagmus**	NR	NR	NR	✓	NR	✗	✓
**Sensorineural hearing loss**	NR	✓	✓	NR	NR	✗<br>-Clinically intact hearing	✓
**Respiratory failure**	✓<br>-Apnea<br>-Respiratory insufficiency	✓<br>-Apnea<br>-Respiratory insufficiency	NR	NR	NR	✓<br>-Respiratory insufficiency	✓<br>-Respiratory insufficiency
**Contractures**	✗	✗	NR	NR	NR	✓<br>-Distal upper and lower limb	✓<br>-Lower limb
**Other**	-Ptosis			-Dysmetria (‘cerebellar syndrome’)<br>-Retinopathy (abnormal ERG)		-Optic atrophy<br>-Bulbar dysfunction<br>-Thoracolumbar scoliosis	-Optic atrophy<br>-Congenital glaucoma<br>-Bulbar dysfunction<br>-Thoracolumbar scoliosis
**Extra-neurological features**							
**Respiratory infections**	✗	✓	✓	NR	NR	✓	✓
**Feeding difficulties**	✓	✓	✓	✓	✓	✓<br>-Gastrostomy	✓<br>-Gastrostomy
**Other**	-Aortic valve insufficiency (mild)	-Hypospadias				-GERD<br>-Premature pubarche	-Acetabular dysplasia<br>-Cryptorchidism (right)
**Investigations**							
**Biochemical investigations**	**Blood Lactate:**<br>-Mild ↑ (x 2 occasions)<br><br>**Blood other:**<br>-CK normal to mild ↑<br><br>**CSF Lactate:**<br>-NR	**Blood Lactate:**<br>-No abnormalities<br><br>**CSF Lactate:**<br>-NR	**Blood Lactate:**<br>-No abnormalities<br><br>**CSF Lactate:**<br>-NR	**Blood Lactate:**<br>-Single episode mild ↑; multiple normal samples<br><br>**CSF Lactate:**<br>-NR	**Blood Lactate:**<br>-No abnormalities<br><br>**CSF Lactate:**<br>-NR	**Blood Lactate:**<br>-No abnormalities<br><br>**Blood other:**<br>-CK normal<br>-Ammonia normal<br>-Acylcarnitine profile normal<br><br>**CSF Lactate:**<br>-No abnormalities	**Blood Lactate:**<br>-Mild ↑ in fewer than 15% of samples assayeds<br><br>**Blood other:**<br>-CK normal<br>-Ammonia normal<br>-Acylcarnitine profile normal<br><br>**CSF Lactate:**

	**Urinary Organic acids:**<br>-No abnormalities	**Urinary Organic acids:**<br>-Lactate (mild ↑)	**Urinary Organic acids:**<br>--No abnormalities	**Urinary Organic acids:**<br>-No abnormalities	**Urinary Organic acids:**<br>--No abnormalities	**Urinary Organic acids:**<br>--No abnormalities	-Not performed<br><br>**Urinary Organic acids:**<br>-Krebs cycle intermediates on single sample; multiple normal samples
**Neuroimaging**	**MRI Brain:**<br>-T2 hyperintensities within basal ganglia and thalamus (bilateral; small and transient)<br>-Diffusion restriction within the dorsal mesencephalon<br><br>**1H-MRS:**<br>-No abnormalities	**MRI Brain:**<br>-No abnormalities	**MRI Brain:**<br>-Enlarged ventricles and mega-cisterna magna<br><br>**1H-MRS:**<br>-No abnormalities	**CT Brain:**<br>-Increased signal within Basal Ganglia (bilateral)<br><br>**MRI Brain:**<br>-No abnormalities	**MRI Brain:**<br>-No abnormalities	**MRI Brain:**<br>-Cerebral atrophy with ex-vacuo dilatation of ventricles<br>-T2 signal normal<br><br>**MRI Spine:**<br>-No abnormalities	**MRI Brain:**<br>-Cerebral atrophy<br>-Optic nerve atrophy<br>-T2 signal normal<br><br>**1H-MRS:**<br>-NAA ↓ (basal ganglia)<br>-No additional abnormalities<br><br>**MRI Spine:**<br>-No abnormalities
**EEG**	NR	NR	NR	NR	NR	**Inter-ictal:**<br>-No abnormalities<br><br>**Ictal:**<br>-Generalized epileptiform discharges (seizure)	**Inter-ictal:**<br>-Independent, asynchronous multifocal spike and spike-wave epileptiform discharges
**Electromyogram (EMG) and nerve conduction studies (NCS)**	**EMG:**<br>-Single Fiber EMG showed increased jitter in the left orbicularis oculi - suggestive of a neuromuscular transmission disorder.	NR	NR	NR	NR	**EMG**:<br>-Large amplitude motor unit action potentials consistent with denervation<br><br>**NCS:**<br>-Reduced amplitude of motor and sensory action potentials and slowed conduction velocities in all nerves screened. Consistent with an axonal sensorimotor polyneuropathy	**EMG**:<br>-Large amplitude motor unit action potentials consistent with denervation<br><br>**NCS:**<br>-Reduced amplitude of motor and sensory action potentials and slowed conduction velocities in all nerves screened. Consistent with an axonal sensorimotor polyneuropathy
**Evoked potentials:**	NR	**BSER**:<br>-Abnormal peak pattern and neural conduction to brainstem	NR	**ERG:**<br>-Abnormal<br><br>**VEP:**<br>-Abnormal	NR	Not done	**BSER**:<br>-Only wave 1 clearly recorded with subsequent components poorly developed; features consistent with severe proximal auditory nerve or brainstem pathology

							**ERG:**<br>-Normal<br><br>**VEP:**<br>-Flash VEP's were diffuse and low amplitude<br>-Pattern reversal VEP's not recordable<br>below 210′ consistent with substantially reduced visual function.
**Muscle biopsy:**	**Histopathological:**<br>-Small muscle fibers with myopathological features<br><br>**Respiratory Chain Enzymes:**<br>-NR	**Histopathological:**<br>-Small muscle fibers<br><br>**Respiratory Chain Enzymes:**<br>-Complex I deficiency	**Histopathological:**<br>-NR<br><br>**Respiratory Chain Enzymes:**<br>-Complex I deficiency	**Histopathological:**<br>-NR<br><br>**Respiratory Chain Enzymes:**<br>-Complex I deficiency	NR	**Histopathological:**<br>-Small and large fibre atrophy with fibre type disproportion<br>-Adipose tissue infiltration<br>-NADH: normal mitochondrial distribution<br>-COX: normal<br>-Gomori: no RRF<br><br>**Electron microscopy:**<br>-Not done<br><br>**Respiratory Chain Enzymes:**<br>-Inconclusive[(4)]	**Histopathological:**<br>-Small and large fibre atrophy with fibre type disproportion<br>-Adipose tissue infiltration<br>-NADH: normal mitochondrial distribution<br>-COX: normal<br>-Gomori: no RRF<br><br>**Electron microscopy:**<br>-Normal mitochondrial number<br>-Normal mitochondrial morphology<br><br>**Respiratory Chain Enzymes:**<br>-Not done

Legend: w = week(s), m = month(s), y = year(s), NR = Not reported, ERG = Electroretinogram, GERD = Gastroesophageal Reflux Disease, ↑ = increase, ↓ = decrease, CK = Creatine Kinase, CSF = cerebrospinal fluid, MRI = Magnetic Resonance Imaging, 1H-MRS = Magnetic Resonance Proton Spectroscopy, NAA = N-acetylaspartate, CT = Computed Tomography, EEG = Electroencephalogram, EMG = Electromyogram, NCS = Nerve conduction studies, BSER = Brainstem evoked response (audiometry), VEP = Visual Evoked Responses, NADH = Nicotinamide Adenine Dinucleotide, COX = Cytochrome oxidase

Note:

1. Subject alive at 4 years of age (at time of publication, 2017).
2. Neurological Features: includes neuro-ophthalmological and non-neurological ophthalmological features.
3. Dysautonomia: reported symptoms of tachycardia, diaphoresis and unexplained fever
4. Respiratory Chain Enzyme analysis proved inconclusive; presumed secondary to increased adipose tissue infiltration.

Supplementary Table 2. Acceptor site usage prediction: chr3:119,234,705_119,234,706AG

Program	WT	Variant	Interpretation	Reference
**SpliceAI**	N/A	0.2	Likely acceptor site gain	Jaganathan K, Kyriazopoulou Panagiotopoulou S, McRae JF, Darbandi SF, Knowles D, Li YI, et al. Predicting Splicing from Primary Sequence with Deep Learning. Cell. 2019 Jan 24;176(3):535-548.e24.
**Splice Rover**	0.856	0.906	Gain in functionality of existing splice acceptor	Zuallaert J, Godin F, Kim M, Soete A, Saeys Y, De Neve W. SpliceRover: interpretable convolutional neural networks for improved splice site prediction. Bioinformatics. 2018 Dec 15;34(24):4180–8.
**DSSP**	0.760	0.848	Gain in functionality of existing splice acceptor	Naito T. Human Splice-Site Prediction with Deep Neural Networks. J Comput Biol. 2018 Aug;25(8):954–61.

Supplementary Table 3. Primer and SSO sequences

Name	Number	5' → 3'
TIMMDC1Ex3F1	P442	ACGAGGCTTCATTCGTTATG
TIMMDC1Ex4R1	P443	CATTCAGACTAGTGTTCACTGTG
TIMMDC1Ex5F1	P444	TGGCATAATTGGAGCCTTGC
TIMMDC1Int5F1	P445	GGATTAGGCAGGATATTTGGG
TIMMDC1Int5F2	P446	CCCCTTTGTCTACCTCCTTTG
TIMMDC1Ex6R1	P448	ATGGAGTGCCTTTCGATCCTT
TIMMDC1 SSO1		/52MOErC/*/i2MOErT/*/i2MOErA/*/i2MOErG/*/i2MOErT/*/i2MOErC/*/i2MOErA/*/i2MOErG/*/i2MOErA/*/i2MOErC/*/i2MOErA/*/i2MOErA/*/i2MOErA/*/**i2MOErC**/*/i2MOErA/*/i2MOErC/*/i2MOErC/*/i2MOErA/*/i2MOErA/*/32MOErC/
TIMMDC1 SSO2		/52MOErG/*/i2MOErT/*/i2MOErC/*/i2MOErA/*/i2MOErG/*/i2MOErA/*/i2MOErC/*/i2MOErA/*/i2MOErA/*/**i2MOErA**/*/i2MOErC/*/i2MOErA/*/i2MOErC/*/i2MOErC/*/i2MOErA/*/i2MOErA/*/i2MOErC/*/i2MOErT/*/i2MOErA/*/32MOErA/
NC5 SSO		/52MOErG/*/i2MOErC/*/i2MOErG/*/i2MOErA/*/i2MOErC/*/i2MOErT/*/i2MOErA/*/i2MOErT/*/i2MOErA/*/i2MOErC/*/i2MOErG/*/i2MOErC/*/i2MOErG/*/i2MOErC/*/i2MOErA/*/i2MOErA/*/i2MOErT/*/i2MOErA/*/i2MOErT/*/32MOErG/

For example for SSO1: 52MOErC, 5' 2-MethoxyEthoxy MeC; 32MOErC, 3' 2-MethoxyEthoxy MeC; i2MOErT, Int 2-MethoxyEthoxy T; i2MOErA, Int 2-MethoxyEthoxy A; i2MOErG, Int 2-MethoxyEthoxy G; i2MOErC, Int 2-MethoxyEthoxy MeC; r_/*/ e.g. rA/*/, Phosphorothioated RNA bases

Figure 8a

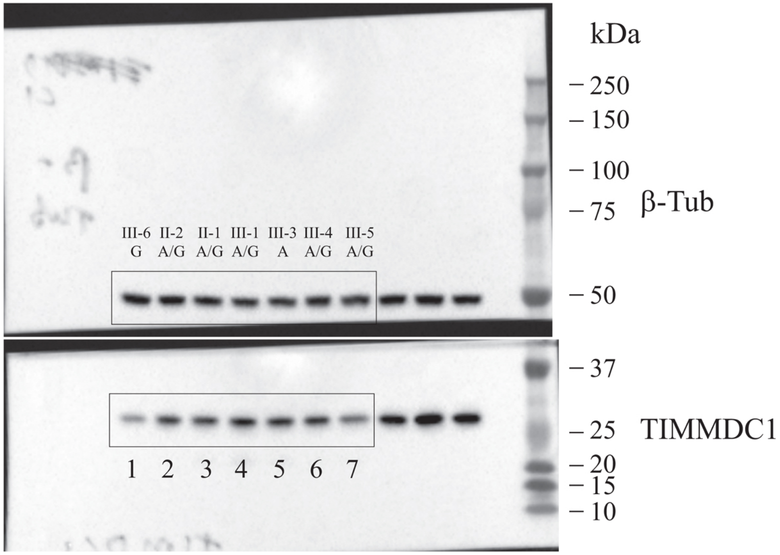

Figure 8b

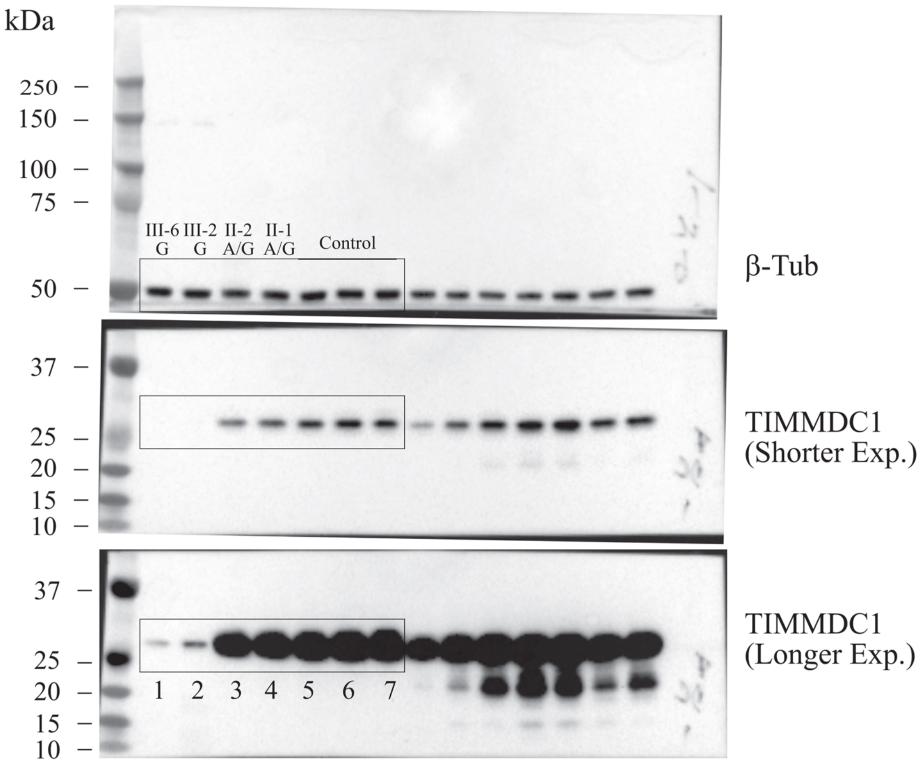

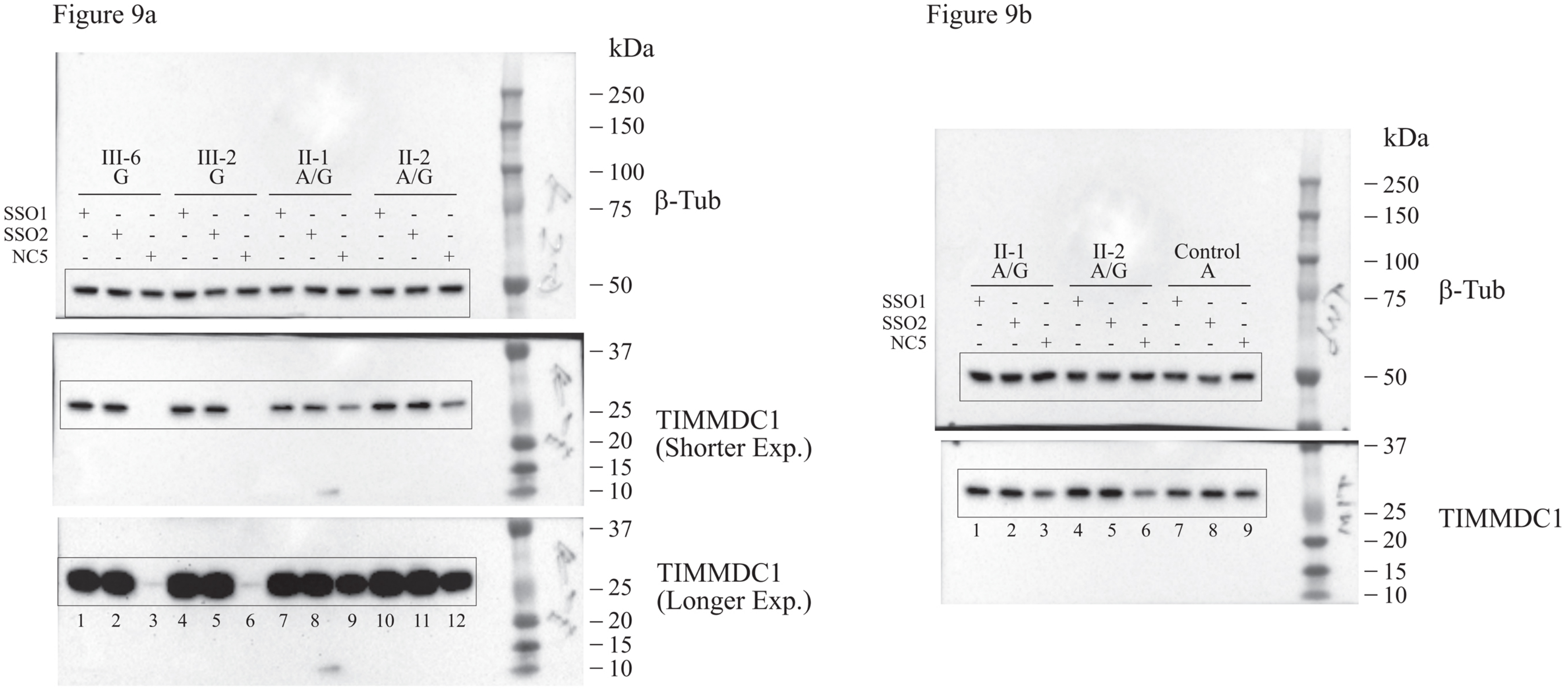

Figure 9a
Figure 9b
kDa
250
150
100
75
50
37
25
20
15
10
β-Tub
TIMMDC1 (Shorter Exp.)
TIMMDC1 (Longer Exp.)
TIMMDC1
III-6 G
III-2 G
II-1 A/G
II-2 A/G
Control A
SSO1
SSO2
NC5
1 2 3 4 5 6 7 8 9 10 11 12
1 2 3 4 5 6 7 8 9
