# Supplementary material for: Oligonucleotide correction of an intronic TIMMDC1 variant in cells of patients with severe neurodegenerative disorder
Source: NPJ Genom Med. 2022 Jan 28;7:9. doi: 10.1038/s41525-021-00277-7 (PMC8799713; doi:10.1038/s41525-021-00277-7)
Supplement: Supplementary file 2 — Reporting Summary [file 41525_2021_277_MOESM2_ESM.pdf]

nature portfolio

Corresponding author(s): Prof. Jozef Gecz

Last updated by author(s): October 12 , 2021

Reporting Summary

Nature Portfolio wishes to improve the reproducibility of the work that we publish. This form provides structure for consistency and transparency in reporting. For further information on Nature Portfolio policies, see our Editorial Policies and the Editorial Policy Checklist.

Statistics

For all statistical analyses, confirm that the following items are present in the figure legend, table legend, main text, or Methods section.

n/a	Confirmed	
☐	☒	The exact sample size (*n*) for each experimental group/condition, given as a discrete number and unit of measurement
☐	☒	A statement on whether measurements were taken from distinct samples or whether the same sample was measured repeatedly
☐	☒	The statistical test(s) used AND whether they are one- or two-sided<br>*Only common tests should be described solely by name; describe more complex techniques in the Methods section.*
☒	☐	A description of all covariates tested
☒	☐	A description of any assumptions or corrections, such as tests of normality and adjustment for multiple comparisons
☐	☒	A full description of the statistical parameters including central tendency (e.g. means) or other basic estimates (e.g. regression coefficient) AND variation (e.g. standard deviation) or associated estimates of uncertainty (e.g. confidence intervals)
☐	☒	For null hypothesis testing, the test statistic (e.g. *F*, *t*, *r*) with confidence intervals, effect sizes, degrees of freedom and *P* value noted<br>*Give P values as exact values whenever suitable.*
☒	☐	For Bayesian analysis, information on the choice of priors and Markov chain Monte Carlo settings
☒	☐	For hierarchical and complex designs, identification of the appropriate level for tests and full reporting of outcomes
☒	☐	Estimates of effect sizes (e.g. Cohen's *d*, Pearson's *r*), indicating how they were calculated

Our web collection on statistics for biologists contains articles on many of the points above.

Software and code

Policy information about availability of computer code

Data collection

No custom code was used for data collection.

Data analysis

No custom code was generated.
The following commercial or open source software was used in analysis:
STAR v2.7.3a
FeatureCounts v1.6.3
edgeR v3.24.3
MitoCarta 3.0
DSSP
SpliceAI
IGV v2.7.2
ANNOVAR (2019 Mar23)

Our statistical approach to quantitative proteomics analyses employing similar instrumentation and methods was performed as in our previous works, that is:
Helman G, et al. Multiomic analysis elucidates Complex I deficiency caused by a deep intronic variant in NDUFB10. Hum Mutat. 2021 Jan;42(1):19-24,
Lake NJ, et al. Biallelic Mutations in MRPS34 Lead to Instability of the Small Mitoribosomal Subunit and Leigh Syndrome.Am J Hum Genet. 2017 Aug 3;101(2):239-254, or
Stroud DA, et al. Accessory subunits are integral for assembly and function of human mitochondrial complex I. Nature. 2016 Oct 6;538(7623):123-126.

For manuscripts utilizing custom algorithms or software that are central to the research but not yet described in published literature, software must be made available to editors and reviewers. We strongly encourage code deposition in a community repository (e.g. GitHub). See the Nature Portfolio guidelines for submitting code & software for further information.

Data

Policy information about availability of data

All manuscripts must include a data availability statement. This statement should provide the following information, where applicable:

- Accession codes, unique identifiers, or web links for publicly available datasets
- A description of any restrictions on data availability
- For clinical datasets or third party data, please ensure that the statement adheres to our policy

Any raw sequence dataset or analyses related to this study are available from the corresponding author upon request, subject to human research ethics approvals.

Field-specific reporting

Please select the one below that is the best fit for your research. If you are not sure, read the appropriate sections before making your selection.

☒ Life sciences ☐ Behavioural & social sciences ☐ Ecological, evolutionary & environmental sciences

For a reference copy of the document with all sections, see nature.com/documents/nr-reporting-summary-flat.pdf

Life sciences study design

All studies must disclose on these points even when the disclosure is negative.

Sample size	Fibroblasts from two affected children, unaffected siblings, parents and unrelated (controls) from individuals with neurodevelopmental disorders (n = 171) or their unaffected family members (n = 107) were used.
Data exclusions	Variants which were unable to be validated by an orthogonal method are not reported.
Replication	Variant reported was validated by PCR/Sanger sequencing of genomic DNA and RT-qPCR/Sanger sequencing on patient (2x), parents (2x) and unrelated control fibroblast RNAs.
Randomization	All samples were tested, randomization was not necessary.
Blinding	Blinding was not required as all samples tested were from affected individuals, unaffected siblings and parents.

Reporting for specific materials, systems and methods

We require information from authors about some types of materials, experimental systems and methods used in many studies. Here, indicate whether each material, system or method listed is relevant to your study. If you are not sure if a list item applies to your research, read the appropriate section before selecting a response.

Materials & experimental systems

n/a	Involved in the study	
☐	☒	Antibodies
☐	☒	Eukaryotic cell lines
☒	☐	Palaeontology and archaeology
☒	☐	Animals and other organisms
☐	☒	Human research participants
☐	☒	Clinical data
☒	☐	Dual use research of concern

Methods

n/a	Involved in the study	
☒	☐	ChIP-seq
☒	☐	Flow cytometry
☒	☐	MRI-based neuroimaging

Human research participants

Policy information about studies involving human research participants

Population characteristics	All cases in this cohort met internationally accepted inclusion criteria for diagnosis a novel form of neuromuscular disorder.

Recruitment	Cases were recruited through Women's and Children's Health Network, South Australia, Australia.
Ethics oversight	The study was approved by the Women's and Children's Health Network human Human research Research Ethics committee Committee (HREC 2361/3/2023) and informed written consents were obtained for all individuals on whom genetic testing and/or molecular investigations were performed.

Note that full information on the approval of the study protocol must also be provided in the manuscript.
